# Dispersive forces and resisting spot welds by alternative homolog conjunction govern chromosome shape in *Drosophila* spermatocytes during prophase I

**DOI:** 10.1371/journal.pgen.1010327

**Published:** 2022-07-27

**Authors:** Luisa Vernizzi, Christian F. Lehner

**Affiliations:** Department of Molecular Life Science (DMLS), University of Zurich, Zurich, Switzerland; College de France CNRS, FRANCE

## Abstract

The bivalent chromosomes that are generated during prophase of meiosis I comprise a pair of homologous chromosomes. Homolog pairing during prophase I must include mechanisms that avoid or eliminate entanglements between non-homologous chromosomes. In *Drosophila* spermatocytes, non-homologous associations are disrupted by chromosome territory formation, while linkages between homologous chromosomes are maintained by special conjunction proteins. These proteins function as alternative for crossovers that link homologs during canonical meiosis but are absent during the achiasmate *Drosophila* male meiosis. How and where within bivalents the alternative homolog conjunction proteins function is still poorly understood. To clarify the rules that govern territory formation and alternative homolog conjunction, we have analyzed spermatocytes with chromosomal aberrations. We examined territory formation after acute chromosome cleavage by Cas9, targeted to the dodeca satellite adjacent to the centromere of chromosome 3 specifically in spermatocytes. Moreover, we studied territory organization, as well as the eventual orientation of chromosomes during meiosis I, in spermatocytes with stable structural aberrations, including heterozygous reciprocal autosomal translocations. Our observations indicate that alternative homolog conjunction is applied in a spatially confined manner. Comparable to crossovers, only a single conjunction spot per chromosome arm appears to be applied usually. These conjunction spots resist separation by the dispersing forces that drive apart homologous pericentromeric heterochromatin and embedded centromeres within territories, as well as the distinct chromosomal entities into peripheral, maximally separated territories within the spermatocyte nucleus.

## Author summary

Already the primordial eukaryote appears to have used meiosis for sexual reproduction, because this sophisticated process follows a canonical program in lineages ranging from unicellular organisms to plants and animals. The maternal and paternal copies of a particular chromosome, i.e., the homologs, are first physically linked into a bivalent before the first meiotic division. Linkage is essential for error-free chromosome segregation. In canonical meiosis, linkage is achieved by crossovers. These are regulated so that each chromosome pair is linked, but only by very few crossovers. Surprisingly, crossovers are absent during meiosis in males of the fruit fly *Drosophila melanogaster*. Instead, an alternative homolog conjunction system is used. It is not yet clear how this functions. Here, we demonstrate that the alternative chromosome glue appears to be applied in a locally restricted manner rather than all along the paired homologs. Just two spots of glue appear to conjoin the two homologous chromosomes usually, with one spot linking the left and another the right chromosome arm. Thus, number and location of linkages appear to be similar as crossovers, raising the possibility of mechanistic similarities in the establishment of the two distinct types of homolog linkage.

## Introduction

At the onset of mitotic and meiotic divisions, interphase chromatin is condensed into chromosomes for segregation by spindles. Chromosomes are comprised of two linked entities, homologous chromosomes in the first meiotic division (M I) and sister chromatids in the second meiotic division (M II) and in mitosis. Each of the two entities assembles a kinetochore (KT) for attachment to spindle microtubules (MTs). The correct amphitelic integration of chromosomes into bipolar spindles involves tension sensing [1]. Pulling forces exerted by kinetochore microtubules (kMTs) generate mechanical tension at KTs of bi-oriented chromosomes, thereby stabilizing the interactions between kMTs and KTs. In contrast, KTs of chromosomes with incomplete (monotelic) or erroneous (syntelic or merotelic) orientation within the spindle experience less tension and remain unstable. Over time, therefore, correct bi-polar attachments prevail, allowing silencing of the M phase checkpoint and progression into anaphase by elimination of the linkage between the chromosomal entities. Appropriate linkages between chromosomal entities are therefore crucial for faithful chromosome segregation. The structure of these linkages must allow their rapid and complete destruction at the metaphase to anaphase transition, or else anaphase bridges arise. Absence of linkage at the onset of M phase as well as presence of inappropriate linkages with other chromosomes interferes with regular chromosome bi-orientation. In case of M I, avoidance of inappropriate connections between chromosomes is particularly challenging. While sister chromatids arise in immediate spatial proximity through DNA replication that is coupled with establishment of cohesin-mediated sister cohesion, homologous chromosomes are at random positions when they have to find each other for pairing into bivalents before M I. Hence, the pairing process can lead to entanglement between bivalents. The resulting mechanical coupling between bivalents is predicted to compromise regular biorientation, as irregular KT attachments might also experience tension.

During canonical meiosis, initial pairing and transient synapsis of homologous chromosomes is succeeded by maintenance of homolog linkage by crossovers (COs) in combination with distal sister chromatid cohesion. In *Drosophila* spermatocytes, however, synaptonemal complex formation and meiotic recombination do not occur. During this achiasmate meiosis, homolog linkage is maintained by an alternative homolog conjunction (AHC) system instead of COs [2]. So far, four AHC proteins have been identified [3–5]. The initial chromosome pairing in early spermatocytes does not depend on these proteins. However, they are required for maintenance of homolog conjunction in bivalents during spermatocyte maturation until onset of anaphase I. One of the four proteins (TEF) is only required for AHC in autosomal bivalents. The other three (MNM, SNM and UNO) are also essential in case of the sex chromosome bivalent. In *Drosophila*, chromosome (chr) X and chrY do not share extended regions of homology within the euchromatic parts. Thus, sex chromosome pairing in *Drosophila* cannot exploit pseudoautosomal regions as for example in humans. However, rDNA loci are embedded within pericentromeric heterochromatin of both chrX and chrY in *D*. *melanogaster*, and these repetitive loci serve as pairing centers in spermatocytes [6]. MNM, SNM and UNO accumulate predominantly in subnucleolar foci in spermatocytes [3,5]. These foci coalesce into a single prominent dot in between the paired sex chromosomes during chromosome condensation around nuclear envelope breakdown (NEBD) of M I. The molecular details of how the AHC proteins are recruited onto rDNA and how they confer physical linkage between the sex chromosomes are not yet understood. Even less clear is the mode of AHC in case of the autosomal bivalents. While TEF detection has not been possible [4], MNM, SNM and UNO can be observed on autosomal bivalents [3,5]. However, in comparison to the very prominent dot associated with the sex chromosomes, autosomal signals of MNM, SNM and UNO are far weaker. They can be detected as faint spots on autosomal bivalents, which also coalesce into a few dots per bivalent around NEBD I. The intra-chromosomal localization of these autosomal dots is not known. However, genetic analyses have argued strongly against the notion that autosomes include a unique invariant locus for AHC, comparable to the rDNA loci of the sex chromosomes [2,7,8]. Rather, the potential for AHC establishment appears to be widely distributed throughout the euchromatic regions of the autosomes, suggesting the possibility that autosomal AHC might perhaps not just arise at the autosomal dots that are detectable just above background, but also far more widespread in additional chromosomal regions with AHC proteins below the detection limit.

Beyond the intra-chromosomal localization of autosomal AHC, the related issue of how AHC is established exclusively between homologs and not also between hon-homologous autosomes is similarly unresolved. Features like DNA sequences or specific epigenetic codes that are widely distributed but nevertheless restricted to only one particular autosome are not known to exist (except in part for the small dot-like chr4). Even if there were such autosome-specific features for AHC protein recruitment, they would not solve the problem of specificity, since the same AHC proteins are used in all the autosomal bivalents for maintenance of conjunction.

For AHC establishment exclusively between homologs, the intriguing and conspicuous process of chromosome territory formation is likely of great importance. Territory formation ensues in early spermatocytes. In *Drosophila* testes, spermatocytes are derived from germline stem cells. After an asymmetric division of these stem cells, the differentiating spermatogonial daughter cell progresses through mitosis with incomplete cell division. After three additional spermatogonial division cycles with incomplete cytokinesis, clusters of 16 spermatocytes, interconnected by ring canals and enveloped by two somatic cyst cells, progress through a four-day growth period subdivided into six stages (S1 –S6) [9]. Thereafter, M I starts with the rapid completion of chromosome condensation and NEBD initiating prometaphase I. Note that “M I” as used here does not include the spermatocyte growth period. Very early in spermatocytes, in the S1 stage, homolog pairing is completed [10], followed by chromosome territory formation during a few hours at the S2a/b stage transition [9–11]. Before territory formation, the distribution of chromatin in spermatocyte nuclei is relatively uniform. However, a chromocenter is clearly present [11], as in the great majority of cell types in *Drosophila*. The chromocenter arises from co-clustering of the highly repetitive, satellite-rich pericentromeric heterochromatin of all the chromosomes [12]. During territory formation, the chromocenter is disrupted and the bivalents are separated apart within the spermatocyte nucleus [9,11]. DNA staining reveals three major chromosome territories in S3-S6 spermatocytes. One of these contains the chr2 bivalent, another the chr3 bivalent and the third hosts the sex chromosome bivalent. Associated with this chrXY territory is usually also the bivalent of the small dot-like chr4 [9,13,14]. The chrXY4 territory is characterized by a pattern of DNA staining that is distinct from the more homogenous appearance of the large autosomal territories [14,15]. Surprisingly, the disruption of non-homologous chromosome associations during territory formation is followed by disruption of additional chromosomal associations. During S3-S5, the tight pairing of homologs and even sister chromatid cohesion is almost completely abolished, as revealed by lacO/lacI-GFP and FISH analyses [10,13]. It has been proposed, therefore, that establishment of AHC is accurately coordinated in time with territory formation [16]. If AHC were established soon after chromosome territory formation, even non-specific indiscriminate chromatin linkers would achieve appropriate linkage exclusively between homologs. Moreover, AHC would have to be in place before the temporally extended phase of chromatin dispersal during territory maturation in S3-S6. Thereby, AHC could protect against complete disruption of homolog associations and maintain some persistent homolog conjunction until anaphase I. In AHC mutant spermatocytes, homolog pairing is completely destroyed during spermatocyte maturation [3,5]. Temporally delayed expression of AHC proteins from transgenes cannot restore AHC in the mutant spermatocytes [16].

As in case of AHC, the molecular mechanisms that drive territory formation and subsequent unpairing of homologs and sister chromatids during spermatocyte maturation are not understood in detail. However, condensin II proteins are crucial for territory formation [11,17]. Chromatin loop extrusion by condensin II presumably drives axial compaction and disruption of non-homologous associations, homolog pairing and sister cohesion. However, the final separation of chromosome territories is extensive with substantial apparently DNA-free gaps in between. This wide spatial separation cannot be explained by condensin II-mediated loop extrusion alone, implying contributions of other dispersive forces, which were also suggested by the residual chromocenter stretching observed in condensin II null mutants [11].

For further clarification of the rules that govern chromosome territory formation and AHC, as well as their functional significance for *Drosophila* male meiosis, we have analyzed spermatocytes with acute or established chromosomal aberrations. Our observations indicate that the forces, which drive apart chromosomal entities during the stages of territory formation and thereafter, act in an indiscriminate manner. All chromosomal entities without stable linkages in between, including chromosome fragments for instance, are extensively separated apart from each other. Our analysis of spermatocytes heterozygous for reciprocal autosomal translocations suggests that the intra-chromosomal positioning of AHC on autosomes might be analogous to that of COs in canonical meiosis. Linkage of autosomal bivalents at the start of M I in spermatocytes appears to be maintained usually by AHC at only a single spot per chromosome arm. The homologous pericentromeric regions with the embedded centromeres are driven apart and away from the AHC spot by the chromatin dispersive forces. In case of heterozygous translocations, ring-shaped quadrivalents can thereby arise. Segregation of the four mechanically coupled centromeres of quadrivalents was often irregular indicating that inter-bivalent associations indeed compromise regular M I biorientation.

## Results

### Chromosome territories in spermatocytes with compound chromosomes

**Fig 1:**
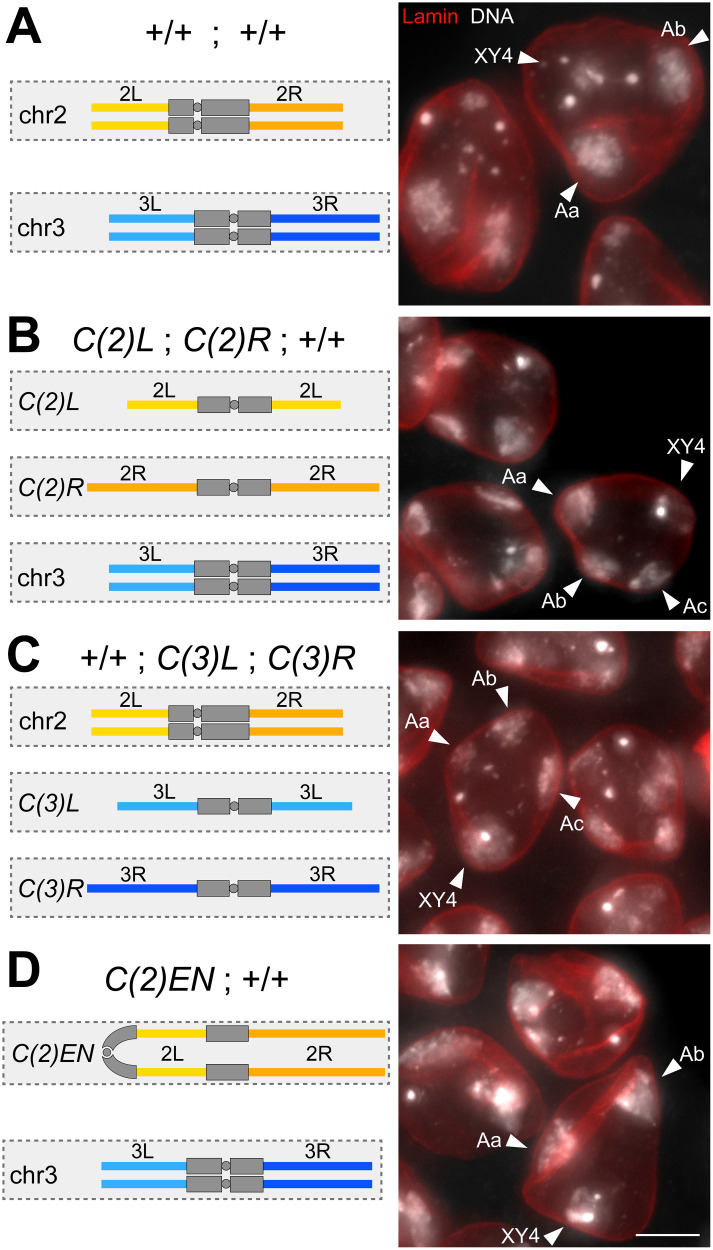
Chromosome territories in spermatocytes with compound chromosomes. (**A-D**) S5 spermatocytes with either the wild-type karyotype (**A**) or the indicated compound chromosomes (**B-D**) were labeled with anti-Lamin Dm0 and a DNA stain. In the schemes on the left, the chromosome territories formed by large autosomes are represented by boxes (dashed outline, light grey shading) and the structure of these autosome is illustrated (euchromatin in color, heterochromatin in dark grey). Arrowheads in the micrographs from squash preparations on the right indicate large autosomal territories (Aa, Ab, Ac) as well as the additional territory (XY4) formed by the sex chromosomes and chr4. Scale bar = 5 μm.

The three major chromosome territories, formed by the sex chromosome bivalent and the large autosomal bivalents of chr2 and chr3, respectively, are readily apparent within the nuclei of wild-type spermatocytes during the stages S3 to S6. The intranuclear positions of the territories are consistent with a separation mechanism that maximizes distances between the major bivalents. The characteristic positioning at the vertices of an approximately equilateral triangle was invariably observed after live imaging of spermatocytes in cysts released from early pupal testes (S1 Movie). In testis preparations obtained with the very widely applied squashing approach, triangular positioning was also apparent in many spermatocytes (Fig 1A), but not in all because of the flattening resulting from squashing. While this long-known characteristic positioning of the major chromosome territories might be directed by a mechanism that simply maximizes the spatial separation between chromosome entities, the bivalent of the small dot-like chr4 complies with this rule only in a minority of the spermatocytes (S1 Movie). In the majority of the S5/6 spermatocytes analyzed after live imaging (72.1%, n = 48 spermatocytes from six independent cysts), the chr4 bivalent was not separated apart but closely associated with the chrXY territory, as previously described [11,13].

To assess the validity of the suggestion that territory organization is governed primarily by a mechanism maximizing distances between chromosomal entities irrespective of their identities, we analyzed spermatocytes with chromosomal aberrations. Surprisingly, while a large number of distinct aberrations have been generated over more than hundred years of *D*. *melanogaster* genetics, the associated chromosome territory organization in spermatocytes before M I has not been reported apparently. Various cytological analyses, including a time-lapse study [18], have characterized aberrant chromosomes during the meiotic divisions. However, territory organization during spermatocyte maturation cannot be inferred necessarily from observations during M I. The number of territories in spermatocytes can be distinct from the number of independent chromosomal masses during prometaphase I. In normal spermatocytes, for example, chr4 and the sex chromosomes are in separate bivalents during prometaphase I, while they are often within a shared territory in spermatocytes until chromosome condensation during NEBD I separates them apart [11,15].

For the characterization of the effects of chromosomal aberrations on territory organization, we started with spermatocytes carrying autosomal compound chromosomes [19,20]. In a first genotype, the two compound chromosomes *C(2L)* and *C(2R)* were present instead of the two normal chr2 homologs. *C(2L)* and *C(2R)* do not share extended homology in their euchromatic regions, but as a result of their derivation, they have some pericentromeric heterochromatic regions in common in all likelihood. However, *C(2L)* and *C(2R)* segregate randomly during male M I [20], and cytological analyses have demonstrated that *C(2L)* and *C(2R)* are not paired during prometaphase I [18,21]. Our testis squash preparations revealed that the nuclei of *C(2L)*; *C(2R)* spermatocytes displayed a characteristic chrXY4 territory and three additional autosomal territories (Fig 1B). Thus, *C(2L)* and *C(2R)* are partitioned into separate territories in spermatocytes. Interestingly, the spacing between the four major territories in *C(2L)*; *C(2R)* spermatocytes appeared to comply with a spacing maximization rule. An analogous territory organization was also observed in spermatocytes with *C(3L)* and *C(3R)* instead of the normal chr3 homologs (Fig 1C). *C(3L)* and *C(3)R* are also known to segregate randomly during male meiosis I [19]. Finally, we analyzed spermatocytes with *C(2)EN*, a more complex compound chromosome [22]. This metacentric has two long arms, each comprised of a chr2L-chr2R fusion (Fig 1D). *C(2)EN* can thus replace both chr2 homologs. Due to its experimental synthesis [22], *C(2)EN* contains regions from the sex chromosomes, in particular from chrY, including a functional rDNA locus. Normally, rDNA loci are only present on the sex chromosomes, serving as meiotic pairing sites [6]. The rDNA locus on *C(2)EN* was hypothesized to cause its biased co-segregation with chrX rather than with chrY during male meiosis [23]. Given the sex chromosome regions on *C(2)EN*, its association with the chrXY territory instead of an independent territory in spermatocytes seemed possible. However, the DNA staining pattern in *C(2)EN* spermatocytes indicated that this compound chromosome was separated into an independent territory that was present in addition to the chrXY4 territory and another major autosome territory (Fig 1D). The three major territories in *C(2)EN* spermatocytes appeared to have a spacing comparable to that in spermatocytes with a normal karyotype.

Overall, our findings with compound chromosomes indicated that partial pericentric heterochromatic homology does not prevent chromosomes from moving apart during territory formation. Moreover, the spacing of chromosome territories observed in spermatocytes with compound chromosomes was consistent with the suggestion that territory formation is directed primarily by a mechanism maximizing distances between chromosomal entities irrespective of their identities.

### Chromosome territory formation after acute chromosome cutting with Cas9

**Fig 2:**
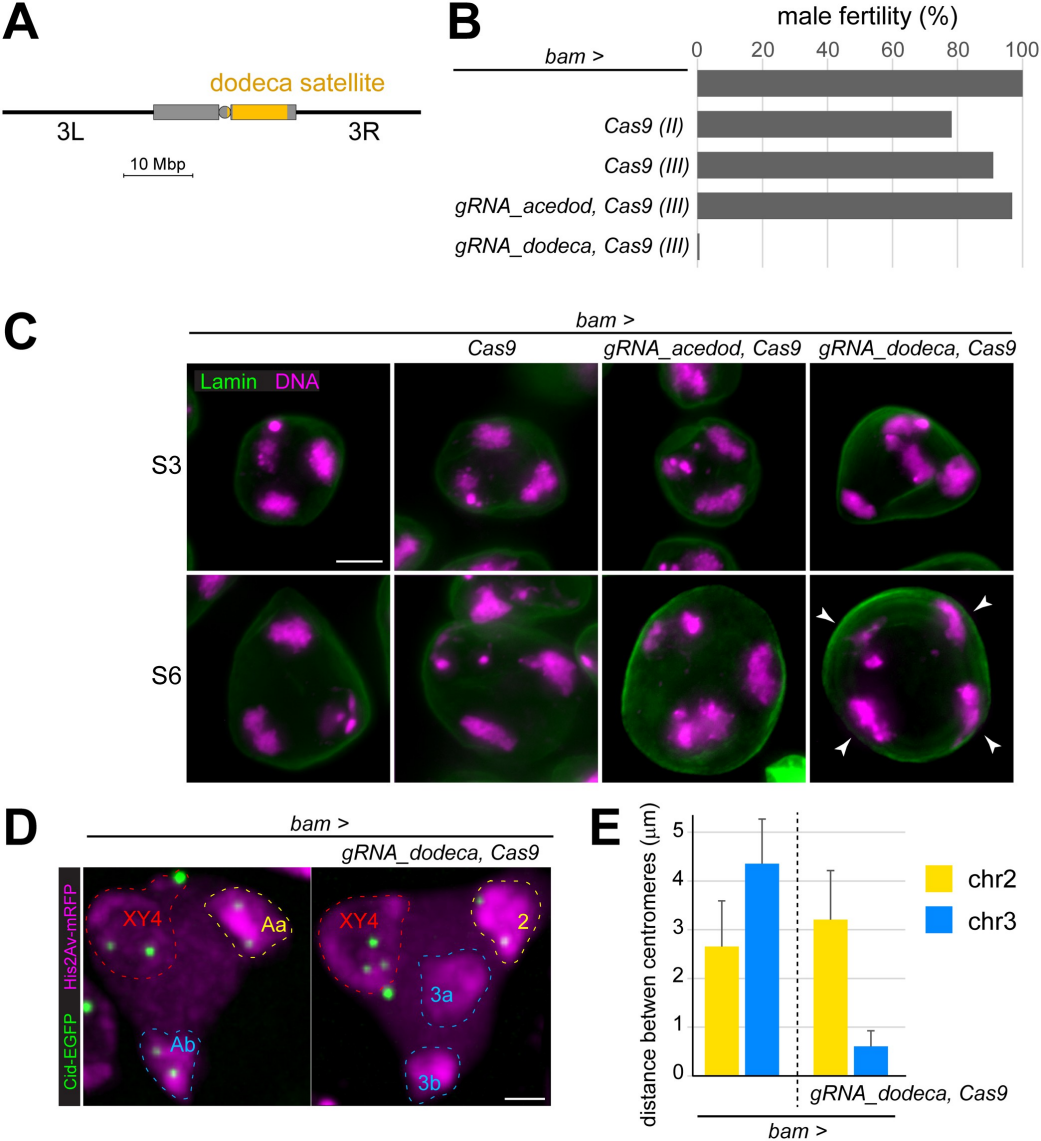
Sub-territory formation after acute cleavage of chr3 by spermatocyte-specific Cas9 targeting to the dodeca satellite repeat. (**A**) Localization of the dodeca satellite. Pericentromeric heterochromatin represented by boxes, centromere by circle. (**B**) Horizontal bars indicate fertility of males, in which *bam-GAL4-VP16* (*bam>*) was used for spermatocyte-specific expression of the indicated UAS transgenes. The fertility of *bam-GAL4-VP16* males without a UAS transgene was set to 100% (top). Co-expression of *Cas9* and a gRNA targeting the dodeca satellite sequence resulted in almost complete sterility (bottom). (**C**) Spermatocytes with *bam-GAL4-VP16* (*bam>*) and the indicated UAS transgenes after labeling testis squash preparations with anti-LaminDm0 and a DNA stain. High magnification views display cells soon after chromosome territory formation (S3) and with maximally separated territories (S6). Four major chromosome territories (arrowheads) instead of the normal three were observed in mature spermatocytes after dodeca satellite cutting. (**D,E**) Chromosome territory organization analyzed by live imaging of spermatocytes expressing *His2Av-mRFP* and *Cenp-A/cid-EGFP*. (**D**) Territories in S6 cells without or with *bam>UAS-Cas9 UAS-gRNA_dodeca* are indicated (dashed lines). Targeting of the dodeca satellite results in the partitioning of the chr3 territory into two sub-territories (3a and 3b). (**E**) Distance between the two Cid-EGFP dots associated with the territories formed by chr2 and chr3, respectively, in the indicated genotypes at NEBD I. Bars represent mean +/- s.d., n = 12 (*control*) and 31 (*dodeca*). Scale bars = 5 μm (C) and 3 μm (D).

For further confirmation that chromosomal identity does not determine territory location, we set out to analyze spermatocytes after acute cleavage of chr3 by directing Cas9 to the dodeca satellite repeat sequence (Fig 2A). The dodeca satellite was estimated to cover at least one million base pairs [24–26]. Its chromosomal location was originally proposed to be pericentromeric, embedding the centromere of chr3 [25], but a more recent centromere characterization [27] indicated a confinement to the right of the centromere (Fig 2A). Fortunately, dodeca repeats are absent from other chromosomes, in contrast to most other more widely distributed satellites [28]. Moreover, while most *Drosophila* satellites are AT-rich, lacking protospacer adjacent motifs (PAMs) for cleavage by CRISPR/Cas9, the dodeca repeat (5’ CCCGTACTCGGT-3’) is GC-rich [26] and includes PAMs (5’-NGG-3’). Therefore, we expected that CRISPR/Cas9 targeting to the dodeca satellite might disconnect the right arm of chr3 from the rest of chr3 (centromere and left arm). The two parts of chr3 generated by Cas9 cleavage should be driven apart into separate territories, if territory formation simply maximizes distances between chromosomal entities irrespective of chromosomal identity.

To induce cleavage of the dodeca satellite specifically in early spermatocytes, we used *bamP-GAL4-VP16* and *UAS-Cas9* in combination with *UAS-gRNA_dodeca* for expression of a gRNA targeting the dodeca satellite sequence. In control experiments, we used *UAS-gRNA_acedod* for expression of the inverted complement of *gRNA_dodeca*. While the control *gRNA_acedod* does not have any perfect-match targets in the known *D*. *melanogaster* genome sequences, *gRNA_dodeca* is predicted to have 8787 prefect-match targets. Two thirds of these predicted *gRNA_dodeca* target sites were located in unmapped contigs, and the remaining third with map locations within the reference genome sequence were all within the centromere-proximal chr3R heterochromatin.

Spermatocyte-specific *Cas9* expression without co-expression of a gRNA transgene did not affect male fertility (Fig 2B); the fertility of *bam>UAS-Cas9* males was comparable to that of control males (*bamP-GAL4-VP16* without a UAS transgene). Similarly, coexpression of *Cas9* and *UAS-gRNA_acedod* did not impair fertility (Fig 2B). However, coexpression of *Cas9* and *UAS-gRNA_dodeca* abolished fertility almost completely (Fig 2B).

The effect of *bam>UAS-Cas9 UAS-gRNA_dodeca* on chromosome territory formation was analyzed with testis squash preparations (Fig 2C). Three genotypes were used as controls: (1.) *bam>* (no UAS transgene), (2.) *bam>UAS-Cas9* and (3.) *bam>UAS-Cas9*, *UAS-gRNA_acedod*. All these control genotypes displayed the normal pattern of three major territories at the S3 stage, i.e., early after territory formation, and also later at the S6 stage, when territories are maximally separated in space (Fig 2C). In contrast, in spermatocytes from *bam>UAS-Cas9 UAS-gRNA_dodeca* males, abnormalities were evident already at the S3 stage and more clearly at the S6 stage when four instead of three major territories were present (Fig 2C). Two of these four territories were not always separated completely but exhibited an interconnecting DNA bridge of variable strength.

As territory organization in spermatocytes was revealed more clearly by live imaging, we introduced the marker transgenes *His2Av-mRFP* and *Cenp-A/cid-EGFP* into the *bam>UAS-Cas9 UAS-gRNA_dodeca* background and analyzed spermatocyte cysts released from dissected early pupal testes. As reported previously [15], the characteristic features of the His2Av-mRFP and Cid-EGFP signals permit an identification of the four distinct bivalents during prometaphase I, and tracking of the His2Av-mRFP signals back in time thus allowed to resolve the distribution and characteristics of identified chromosome territories in late S5 spermatocytes. Thereby, it could be established that the territory abnormalities induced by dodeca satellite targeting concerned chr3, as expected. Two well-separated sub-territories (3a and 3b) were usually displayed (Fig 2D and S2 Movie), instead of the single chr3 territory characteristic of control spermatocytes (Fig 2D and S1 Movie). In contrast, the two other territories (chrXY4 and chr2) were not affected by dodeca satellite targeting.

The centromeres of chr3 were also affected by dodeca satellite targeting. While the chr3 territory normally contains two widely separated Cid-EGFP dots (the homologous centromeres) [15], the two chr3 sub-territories resulting from dodeca satellite targeting showed an irregular association with Cid-EGFP dots. Only one Cid-EGFP dot or two dots with abnormally close spacing were present in either only one of the two sub-territories or in between. In normal control bivalents, the distance between homologous centromeres at NEBD I was around three to four μm in case of both chr2 and chr3 (Fig 2E). In *bam>UAS-Cas9 UAS-gRNA_dodeca* spermatocytes, however, the Cid-EGFP dots associated with chr3 were either not resolved (in 60% of the cells) or closely adjacent (Fig 2E). In contrast, the spacing of the homologous centromeres in the chr2 bivalent was normal after dodeca satellite targeting (Fig 2E).

We conclude that Cas9 targeting to the dodeca satellite resulted in the formation of two chr3 sub-territories that were well separated from each other and from the other bivalents. This phenotype provides strong support for the notion that territories are formed by a mechanism that maximizes distances between chromosomal entities irrespective of their identities.

By time-lapse imaging, progression through M I after dodeca satellite targeting was also analyzed (S1 Text and S3 Movie). This indicated that the two chr3 sub-territories usually had some residual interconnection even when these sub-territories displayed an apparent wide separation within the spermatocyte nucleus before the onset of M I. The interconnections between the chr3 sub-territories were revealed around NEBD I, because chromosome condensation pulled the chr3 sub-territories together (for additional description and discussion of the M I abnormalities see S1 Text).

### Chromosome territory formation in spermatocytes heterozygous for reciprocal translocations between the large autosomes

**Fig 3:**
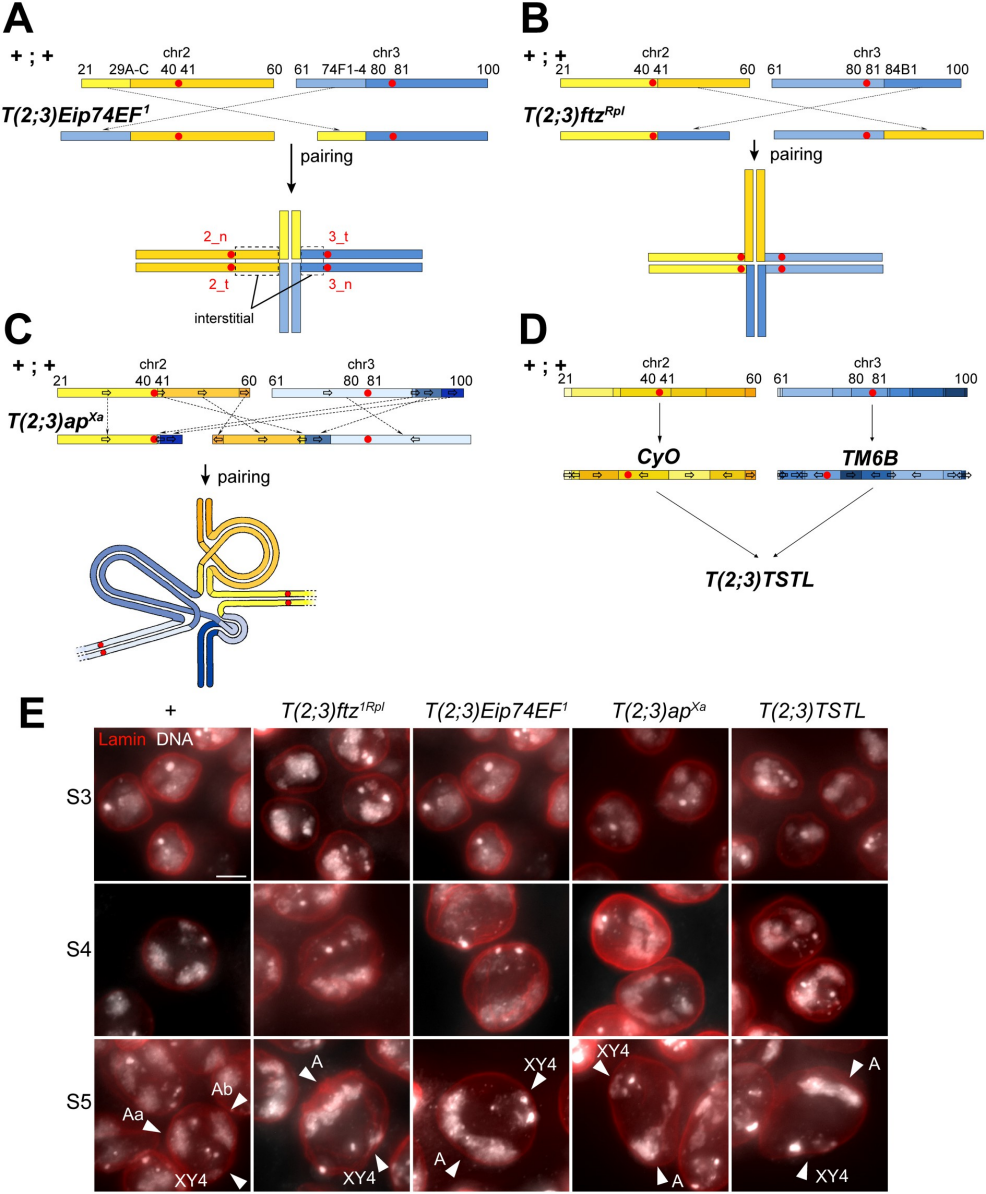
Chromosome territories in spermatocytes heterozygous for reciprocal *T(2;3)* translocations. (**A-D**) Structure of the analyzed reciprocal *T(2;3)* translocations and of the expected pairing into quadrivalents in heterozygous cells. Centromeres are indicated by red circles, and in (A) they are labeled in red type, indicating chromosome number (2 or 3) and presence on either normal (n) or translocation (t) chromosome. The regions between centromeres and translocation breakpoints are designated as interstitial segments. Note that pericentromeric heterochromatin has been omitted. The type I translocations, *T(2;3)Eip74EF*^*1*^ (A) and *T(2;3)ftz*^*Rpl*^ (B), have a single chromosomal translocation break point. In contrast, the type II translocations, *T(2;3)ap*^*Xa*^ (C) and *T(2;3)TSTL* (D), have multiple chromosomal break points because the precursor chromosomes carried inversions. Pairing in case of type II translocation heterozygotes requires complex looping gas illustrated in (C) (adapted from [31]). (**E**) Squash preparations of testes from males, which were either wild-type (+) or heterozygous for the indicated *T(2;3)* translocations, were labeled with anti-Lamin Dm0 and a DNA stain. As illustrated by spermatocytes at the indicated stage, two instead of the normal three chromosome territories were formed in the translocation heterozygotes. Arrowheads indicate large autosomal territories (Aa and Ab in wild-type, and A in translocation heterozygotes) and the territories (XY4) containing the sex chromosomes and chr4. Scale bar = 5 μm.

Among the different structural types of chromosome aberrations, reciprocal autosomal translocations appeared of particular interest for the study of chromosome territories in spermatocytes. The meiotic pairing of homologous chromosomal regions in translocation heterozygotes is predicted to result in a quadrivalent, an association of four chromosomes with two pairs of homologous centromeres (Fig 3A). Quadrivalent formation has been demonstrated cytologically, or inferred from genetic crosses with suitable marker mutations. Cytologically, quadrivalents have been studied most extensively in plants during canonical meiosis [29]. Quadrivalent formation was first reported in maize, with a cross-shape at pachytene and a ring-shape at metaphase I [30]. Later cytological analyses have established that the shape of meiotic translocation chromosomes varies, depending on factors including the size of the involved chromosomes, the positions of breakpoints and centromeres, as well as the number and positions of COs [29]. While quadrivalent formation is the rule, alternative modes of chromosomal associations can result, like the combination of a trivalent and a univalent or of two bivalents. In case of quadrivalents, the conversion from cross to ring shape occurs after SC disassembly during diplotene and depends on COs being present in all four limbs of the cross and absent from the interstitial region (Fig 3A). If one of the limbs is devoid of a CO, a linear chain quadrivalent will arise. Similarly, with two limbs lacking COs, the combination of a trivalent and a univalent, or of two bivalents is formed instead of a single quadrivalent.

Beyond spatial chromosome organization, the pattern of centromere segregation during the first meiotic division can also vary in heterozygotes with different reciprocal translocations. In general, 2:2 segregation of the four associated centromeres is more frequent than 3:1 or 4:0. Segregation 2:2 can result from three distinct orientation patterns, designated as alternate, adjacent-1 or adjacent-2. Alternate segregation results in euploid progeny (see Fig 3A, centromeres 2_n and 3_n away from 2_t and 3_t), while aneuploid progeny results from adjacent-1 (2_n and 3_t away from 2_t and 3_n) and adjacent-2 (2_n and 2_t away from 3_n and 3_t).

In case of the achiasmate meiosis in *D*. *melanogaster* males, autosomal translocation heterozygotes have been studied by genetic marker segregation analyses (for example [31]), but cytological studies do not appear to have been published so far. For our analyses, we selected reciprocal translocations between the two large autosomes, chr2 and chr3. Four distinct *T(2;3)* translocations of two different types were analyzed. The type I translocations, *T(2;3)Eip74EF*^*1*^ and *T(2;3)ftz*^*Rpl*^, were translocations between structurally normal versions of chr2 and chr3 (Fig 3A and 3B). The type II translocations, *T(2;3)ap*^*Xa*^ and *T(2;3)TSTL*, were translocations between structural variants of chr2 and chr3 (Fig 3C and 3D). *T(2;3)ap*^*Xa*^ is a reciprocal translocation derived from the inversion chromosomes *In(2R)Cy* and *In(3R)P* [32] (Fig 3C). The complex nature of *T(2;3)ap*^*Xa*^ is expected to affect the pairing of the homologous sequences of chr2 and chr3. Full pairing into a quadrivalent requires adoption of a highly convoluted structure (Fig 3C) [31]. The second complex translocation, *T(2;3)TSTL*, is the result of a reciprocal exchange between the balancer chromosomes *CyO* and *TM6B*, which carry multiple nested inversions (Fig 3D). The translocation breakpoints of *CyO* and *TM6B* in *T(2;3)TSTL* are not known. However, complete pairing of all homologous regions in *T(2;3)TSTL* heterozygotes would depend on even greater structural convolution than in case of *T(2;3)ap*^*Xa*^.

Testis squash preparations indicated that all four translocations had the same effect on the number of chromosome territories in heterozygous spermatocytes (Fig 3E). In early S2b and S3 spermatocytes, when the three major territories (Aa, Ab, XY4) start to become apparent in control spermatocytes, translocation heterozygotes displayed a pattern of DNA staining without comparably separated territories (Fig 3E). At later stages, during S4 and S5, translocation heterozygotes normally displayed a single large autosomal territory, rather than two major autosomal territories as in the control (Fig 3E). Thus, the potential pairing impediment associated with complex type II compared to type I translocations did not effectively preclude co-segregation of all the large autosomes into a single large autosome territory.

### Compromised disjunction of homologous centromeres in quadrivalent chromosomes formed in *T(2;3)* heterozygous spermatocytes during the first meiotic division

The generation of a single territory containing all the large autosomal homologs in spermatocytes heterozygous for *T(2;3)* translocations provided an opportunity to address the functional significance of chromosome territory formation. This process disrupts the associations between non-homologous chromosomes that are present initially in early spermatocytes, within the chromocenter for example. Thereby, territory formation assures that individualized, mechanically independent bivalents are present for biorientation at the start of M I. The single large autosomal territory in *T(2;3)* heterozygotes models a partial territory formation failure. An eventual condensation of this territory into a single quadrivalent chromosome mass with two pairs of homologous centromeres may compromise the regular 1:1 segregation of homologous centromeres during M I. Chromosome masses with more than a single pair of homologous KTs, like quadrivalents, might allow stabilization of syntelic attachments of two homologous KTs during prometaphase I, if additional KTs of the chromosome mass establish linkage to the opposite spindle pole. Similarly, if a 3:1 orientation were sufficient for KT attachment stabilization, an increased segregation of centromeres at uneven ratios is expected. In the past, analysis of genetic marker segregation into progeny has given limited and indirect insight into chromosome behavior during the meiotic divisions in *Drosophila* males heterozygous for reciprocal autosomal translocations [31,33]. For direct observation, we applied time-lapse imaging.

**Fig 4:**
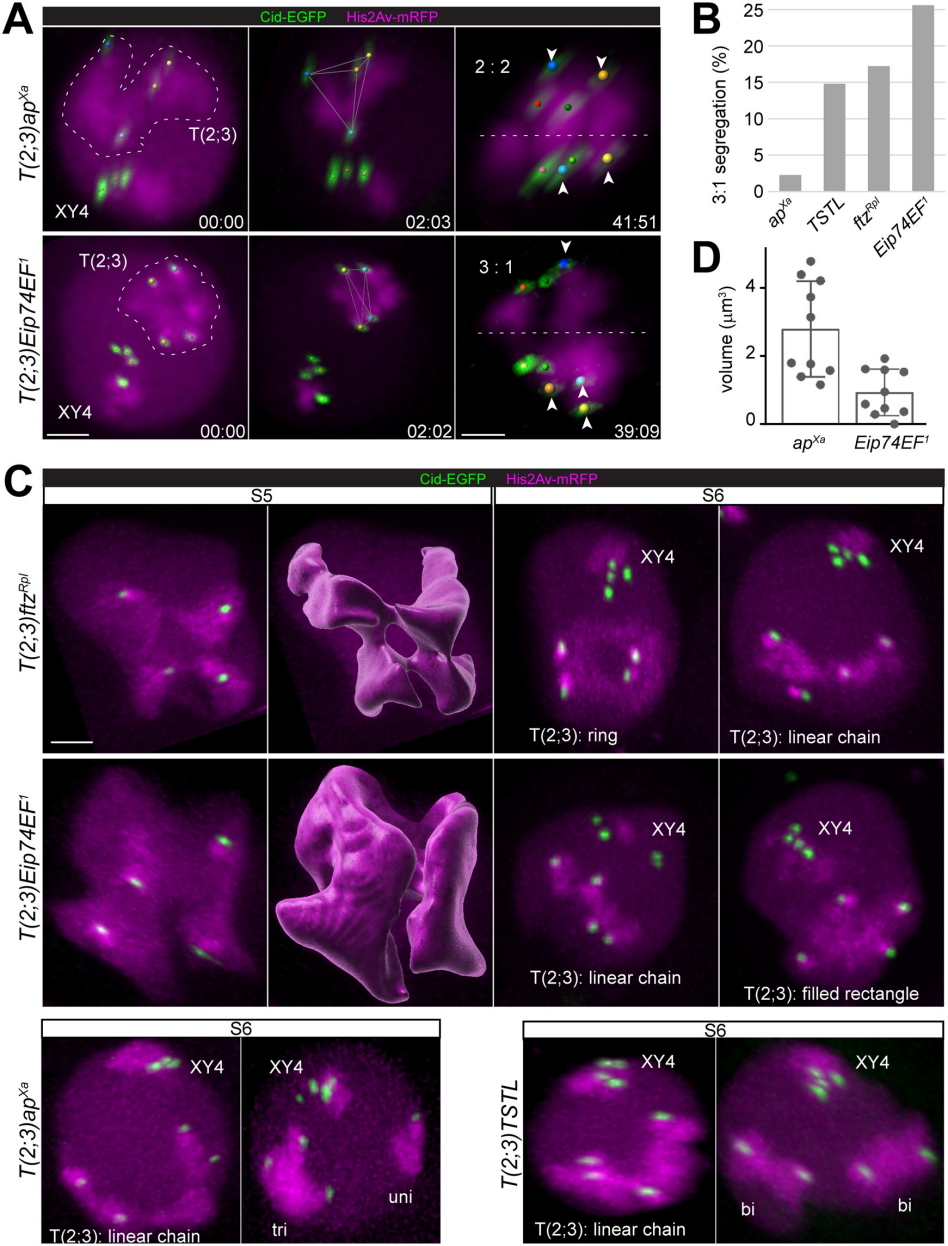
Differences in quadrivalent shape and centromere orientation during M I in distinct *T(2;3)* translocations. Spermatocytes heterozygous for the indicated reciprocal *T(2;3)* translocations were analyzed by time lapse imaging using His2Av-mRFP and Cenp-A/Cid-EGFP as markers. (**A**) Still frames illustrate progression from onset NEBD I until end of metaphase I. The territories formed by the quadrivalent [T(2;3)] and by the other chromosomes (XY4) are indicated in the first frame. Tracked centromeres are marked by spheres with colors indicating the associated chrX and chrY (red), chr4 (green), chr2 and chr3 (yellow and blue). The equatorial plane (dashed lines in the last frame) reveals the segregation pattern of the four quadrivalent centromeres (arrowheads, larger spheres) as 2:2 (A, top) or 3:1 (A, bottom). Time (min:sec) relative to onset NEBD I. (**B**) Bars represent the frequency of 3:1 segregation of the quadrivalent during M I in spermatocytes heterozygous for the indicated *T(2;3)* translocations. n = 44 (*ap*^*Xa*^), 54 (*TSTL*), 58 (*ftz*^*Rpl*^) and 86 (*Eip74EF*^*1*^) spermatocytes from at least seven distinct cysts. (**C**) Spatial organization of quadrivalent chromosome territories in spermatocytes heterozygous for the indicated *T(2;3)* translocations during S5 or after partial chromosome condensation in late S6. For S5, only the part of the nucleus with the quadrivalent is shown next to an isosurface representation of this territory. Quadrivalents (T(2;3) were found to adopt distinct shapes, as indicated. Instead of quadrivalents, some spermatocytes displayed the combination of a trivalent (tri) and an univalent (uni) or of two bivalents (bi). (**D**) The volume of the tetrahedron defined by the four quadrivalent Cid-EGFP dots two minutes after NEBD I (see middle frame in (A)) was measured in spermatocytes heterozygous for the indicated *T(2;3)* translocations. Bars represent mean +/- s.d. (n = 10). Scale bars = 3 μm.

For time-lapse analysis, the *T(2;3)* translocation chromosomes were made heterozygous with structurally normal versions of chr2 and chr3 that carried the *His2Av-mRFP* and *Cenp-A/cid-EGFP* marker transgenes. Time-lapse imaging clearly revealed that the single large autosomal territory was condensed around the time of NEBD I into a quadrivalent, a compact chromosome mass with four associated Cid-EGFP dots (Fig 4A). The orientation of the four centromere dots of the quadrivalents during metaphase I and anaphase I revealed 2:2 segregation in a clear majority of the spermatocytes (Fig 4A and 4B). However, 3:1 segregation was detected as well (Fig 4A and 4B). Interestingly, the frequency of 3:1 segregation varied greatly between the different translocations (Fig 4B). *T(2;3)ap*^*Xa*^ displayed the lowest 3:1 frequency of 2% and *T(2;3)Eip74EF*^*1*^ the highest of 26%.

We conclude that the regular 1:1 segregation of homologous centromeres is compromised when the large autosomal homologs are all associated in a quadrivalent instead of being individualized into two independent bivalents before the onset of M I.

### Translocation-specific differences in quadrivalent shape

Visualization of chromosome territories by live imaging in a more faithful, three-dimensional state compared to the flattened distorted appearance in squash preparations clearly exposed differences in quadrivalent shapes characteristic for the distinct *T(2;3)* translocations (Fig 4C and 4D), potentially contributing to their different 3:1 segregation rates. A most striking difference in quadrivalent territory shape was observed between the two simple translocations, *T(2;3)Eip74EF*^*1*^ and *T(2;3)ftz*^*Rpl*^.

**Fig 5:**
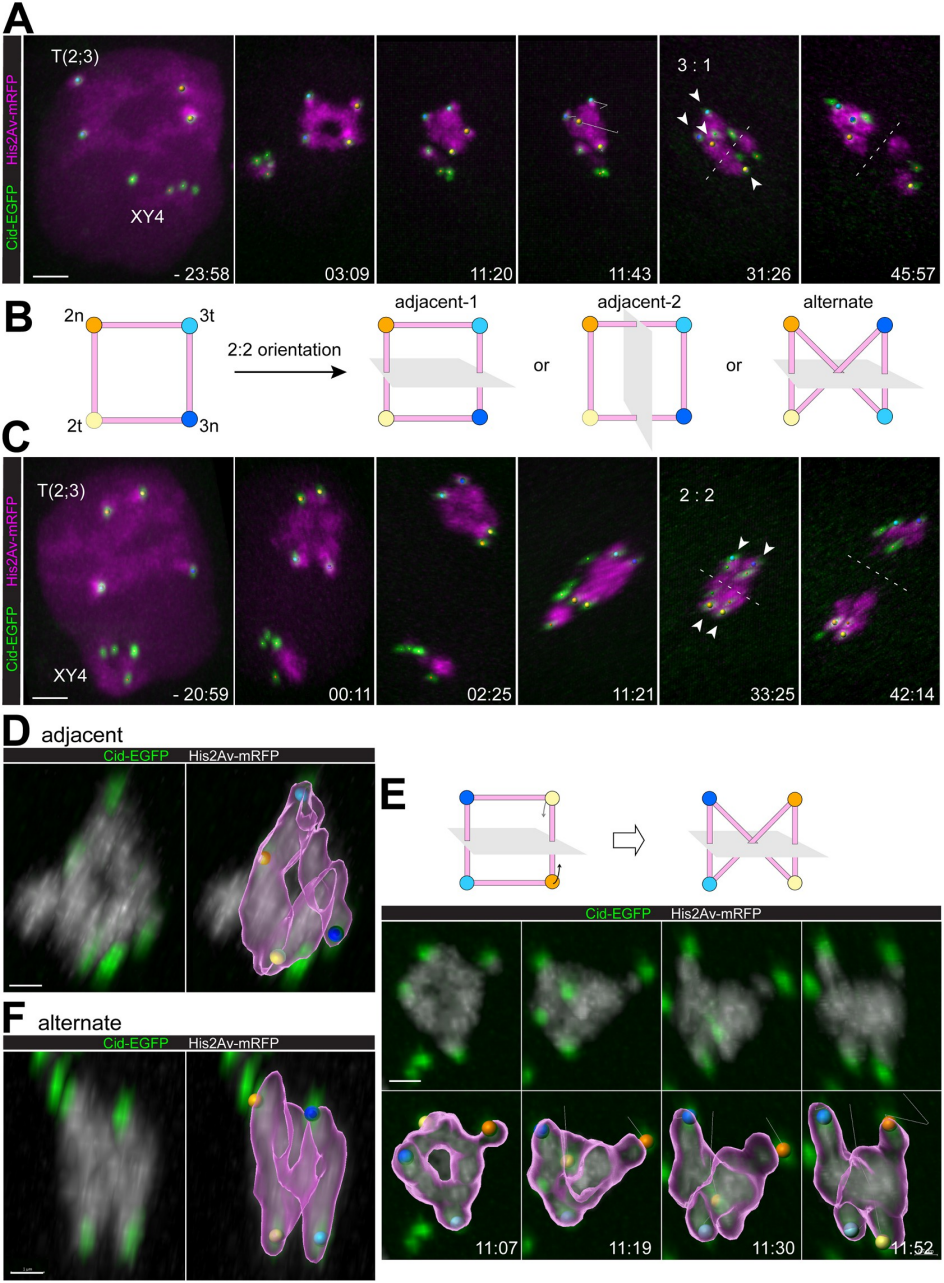
Alternative types of ring quadrivalent segregation during M I. Quadrivalent segregation during M I in spermatocytes heterozygous for *T(2;3)ftz*^*Rpl*^. His2Av-mRFP and Cenp-A/Cid-EGFP were used for time-lapse imaging. Time (min:sec) is indicated relative to the onset of NEBD I. Additional labeling as described (Fig 4A). (**A**) Still frames illustrating 3:1 segregation. (**B**) Schematic illustration of the three distinct modes of 2:2 segregation of the four centromeres of a ring quadrivalent. Centromere labeling as in Fig 4A. Grey planes represent division plane. (**C**) Still frames illustrating 2:2 segregation. (**D,F**) High magnification views of quadrivalents in metaphase I (spindle axis within image plane from top to bottom). The four associated centromeres are bi-oriented in either adjacent (**D**) or alternate (**F**) configuration. His2Av-mRFP isosurface representations of quadrivalent chromatin with centromeres marked by colored spheres are shown on the right side. (**E**) Quadrivalent shape transformation from ring to anti-parallelogram for alternate centromere bi-orientation, as schematically illustrated (top). Scale bars = 3 μm (A,C) and 1 μm (D-E).

The quadrivalent territory in *T(2;3)ftz*^*Rpl*^ heterozygotes was usually ring shaped (Fig 4C, top). During the S5 stage, the ring was composed of four connected lobes, each containing one centromere (Fig 4C, top row, and S4 Movie). The four lobes were separated into peripheral nuclear bulges often with strikingly deep folds in between. Partial condensation and release of chromosomes from the increasingly spherical NE during S6 resulted in a more even ring quadrivalent (Fig 4C, top row, and S5 Movie). During the final rapid chromosome condensation at NEBD I, the ring was further compacted but a central hole often remained detectable during prometaphase I (Fig 5A). Among 29 spermatocytes (from six different cysts), 24 (83%) displayed an obvious ring of chromatin with a central hole when scored around NEBD I. However, among the remaining spermatocytes, some quadrivalents were definitely not ring-shaped, but rather a linear chain of two interconnected bivalents (Fig 4C, top row, and S6 Movie).

In *T(2;3)Eip74EF*^*1*^ heterozygotes, the quadrivalent territory was rarely ring-shaped, in stark contrast to *T(2;3)ftz*^*Rpl*^ heterozygotes. At the onset of NEBD I, only 4 (8%) of the heterozygous *T(2;3)Eip74EF*^*1*^ spermatocytes (n = 51 from 10 distinct cysts) displayed a quadrivalent with a central hole. These holes were smaller than in the ring quadrivalents of *T(2;3)ftz*^*Rpl*^ heterozygotes. The predominant quadrivalent organization in heterozygous *T(2;3)Eip74EF*^*1*^ spermatocytes featured two bivalent-like sub-territories joined via a single interconnecting chromatin region during S5 (Fig 4C, middle row, and S7 Movie). Each of the two interconnected sub-domains contained a pair of widely separated centromeres. The appearance of the centromere-proximal His2Av-mRFP signals were more similar within a sub-domain than between the two sub-domains, indicating that each sub-domain contained a pair of homologous centromeres. Each sub-territory tended to form a separate prominent nuclear bulge with a deep indentation in between. Later during S6, after cell rounding and partial chromosome condensation just before NEBD I, most quadrivalents had a linear chain appearance (Fig 4C, middle row). Linear chains were observed in 33 cells (64%). In 12 cells (24%), the quadrivalent was compacted into a filled rectangle with the four centromeres at the corners (Fig 4C, middle row). It needs to be acknowledged that classification of quadrivalent organization as either linear chain or filled rectangle was ambiguous occasionally because of the similarity between filled rectangles and strongly kinked linear chains (2 of 51 spermatocytes were not classified).

Quadrivalents of spermatocytes heterozygous for the two complex translocations, *T(2;3)ap*^*Xa*^ and *T(2;3)TSTL*, were linear chains in in the large majority (Fig 4C, bottom row). Linear chains, usually bent into horse-shoe shape, were present in 86.8% of the cells in case of *T(2;3)ap*^*Xa*^ (n = 68 cells from 8 distinct cysts) and in 81.1% in case of *T(2;3)TSTL* (n = 53 from 8 distinct cysts) when scoring during S6 and around NEBD I. Only very few quadrivalents were ring-shaped with an obvious central hole over several time frames comparable to *T(2;3)ftz*^*Rpl*^ heterozygotes. Moreover, in case of the ring quadrivalents in complex translocation heterozygotes, one or two of the chromatin connections between the four centromeres were weak unlike the four connections of comparable strength in the quite symmetric rings of *T(2;3)ftz*^*Rpl*^ heterozygotes. In case of the type II translocations, the frequency of ring quadrivalents was 11.8% (*T(2;3)ap*^*Xa*^) and 13.2% (*T(2;3)TSTL*); these numbers represent maxima, as some of the counted putative rings might actually be linear chains. Quadrivalents with a filled-rectangle appearance, as in *T(2;3)Eip74EF*^*1*^ heterozygotes, were not apparent in case of the complex translocations. However, some noteworthy alternatives to quadrivalents were detected. In case of *T(2;3)ap*^*Xa*^, one spermatocyte presented a trivalent and an univalent instead of a quadrivalent (Fig 4C, bottom row). Similarly, among the *T(2;3)TSTL* heterozygous spermatocytes, four spermatocytes displayed two bivalents instead of a quadrivalent (Fig 4C, bottom row).

The quadrivalent shape most relevant for centromere orientation during M I is that at the time when the interactions between KTs and spindle MTs start, i.e. a few minutes after NEBD I [15]. At this time, the quadrivalents in case of *T(2;3)ap*^*Xa*^ and *T(2;3)Eip74EF*^*1*^, which both were predominantly linear chains, were different with regard to the arrangement of the four associated centromeres (Fig 4A and 4D). The volume of the tetrahedron defined by the four Cid-EGFP dots of the quadrivalent (Fig 4A, second time frame) two minutes after the onset of NEBD I was significantly smaller in *T(2;3)Eip74EF*^*1*^ compared to *T(2;3)ap*^*Xa*^ (Fig 4D; p = 0.0014 in t test).

In conclusion, live imaging of chromosome territories in *T(2;3)* heterozygous spermatocytes demonstrated that quadrivalents displayed distinct types of spatial organization. Quadrivalent territories were in shape of a “ring”, a “linear chain” or also of a “filled rectangle”. Moreover, in rare cases, quadrivalents were replaced by the combination of a trivalent and a univalent or of two bivalents. The frequencies of the spatial types of quadrivalent organization were distinct for the different *T(2;3)* translocations.

### Flexibility of quadrivalent chromosomes

While quadrivalent shape might contribute to the distinct rates of 3:1 segregation observed with the four *T(2;3)* translocations, a simple correlation between shape and 3:1 segregation was not obvious. Beyond shape, quadrivalent flexibility during prometaphase I may influence KT orientation within the M I spindle. To explore quadrivalent flexibility, we imaged progression through M I with high spatial and temporal resolution. We focused on *T(2;3)ftz*^*Rpl*^ heterozygous spermatocytes, because distortions of their initially rather regular ring quadrivalents should be well recognizable. Moreover, these ring quadrivalents displayed centromere-proximal His2Av-mRFP signals with characteristics corresponding to those previously described in control spermatocytes [15]. In the controls, the centromere of chr3 was shown to be associated with more pronounced heterochromatic His2Av-mRFP blobs during early prometaphase I compared to chr2 [15]. Accordingly, we assigned identities to the two pairs of Cid-EGFP dots of the ring quadrivalent as centromeres of chr2 and chr3, respectively, in favorable cells with reliable KT tracks. This indicated that the orientation of the four quadrivalent centromeres and the resulting segregation pattern were variable (Fig 5). Moreover, establishment of some centromere orientation patterns were found to be accompanied by extensive distortion of the ring quadrivalent.

In case of 3:1 orientation, the initial ring shape became strongly distorted when one of the four centromeres was eventually pulled over towards the more distant pole and closer to two additional centromeres oriented to this same pole, while the fourth centromere was pulled to the opposite pole (Fig 5A and S8 Movie).

In principle, orientation of the four quadrivalent centromeres in a 2:2 pattern should be possible in three distinct modes (Fig 5B), adjacent-1, adjacent-2 or alternate, and the latter is predicted to be coupled with a strong deformation of the initial ring. As further detailed below, all three modes appeared to occur in case of *T(2;3)ftz*^*Rpl*^ quadrivalents. A statistical analysis of the different 2:2 orientation modes was not attempted because sufficiently reliable KT tracking was feasible only in a minority of spermatocytes, but according to our analyses, the three modes might be comparable in frequency.

Adjacent 2:2 orientation and segregation did not involve severe ring distortions except for stretching along the spindle axis (Fig 5C and S9 Movie). The bi-oriented quadrivalent still presented a central hole in optimally resolved cases (Fig 5D and S10 Movie). In contrast, alternate 2:2 orientation and segregation was accompanied by a shape transformation of the quadrivalent ring to an anti-parallelogram configuration (Fig 5E and S11 Movie). After alternate 2:2 biorientation, the quadrivalents no longer displayed a central hole (Fig 5F and S12 Movie).

Flexibility of the quadrivalent was not only apparent during biorientation of the *T(2;3)ftz*^*Rpl*^ ring quadrivalent, but also for the most compact quadrivalents, the filled rectangles in *T(2;3)Eip74EF*^*1*^ heterozygotes. Distorted quadrivalents after 3:1 orientation were observed in this latter genotype (Fig 4A), as well as cells, in which one pair of centromeres of quadrivalent was re-oriented relative to the other pair during prometaphase I (S1 Fig and S13 Movie).

We add that our analysis of KT tracks during M I in *T(2;3)* heterozygous spermatocytes revealed cases, in which the relatively large mass of quadrivalent chromatin appeared to cause the missegregation of normal bivalents presumably by shielding them from MTs originating from a spindle pole positioned behind the quadrivalent (S1 Fig and S13 Movie).

In conclusion, our time lapse imaging of progression through M I in *T(2;3)ftz*^*Rpl*^ heterozygous spermatocytes indicated that the overall spatial chromosome organization was maintained when the quadrivalent territory was compacted by chromosome condensation into a ring quadrivalent around the time of NEBD I. The four centromeres of the ring quadrivalent remained maximally separated at peripheral positions with chromatin connections in between, generating a ring-like chromosome mass. This spatial arrangement displayed intrinsic stability as well as flexibility. The ring shape was stable unless distorted by forces exerted by spindle MTs on KTs during quadrivalent orientation. These forces resulted in stretching along the spindle axis after adjacent 2:2 orientation, or in folding over after 3:1 or alternate 2:2 orientation.

### Localization of MNM-EGFP on quadrivalents

**Fig 6:**
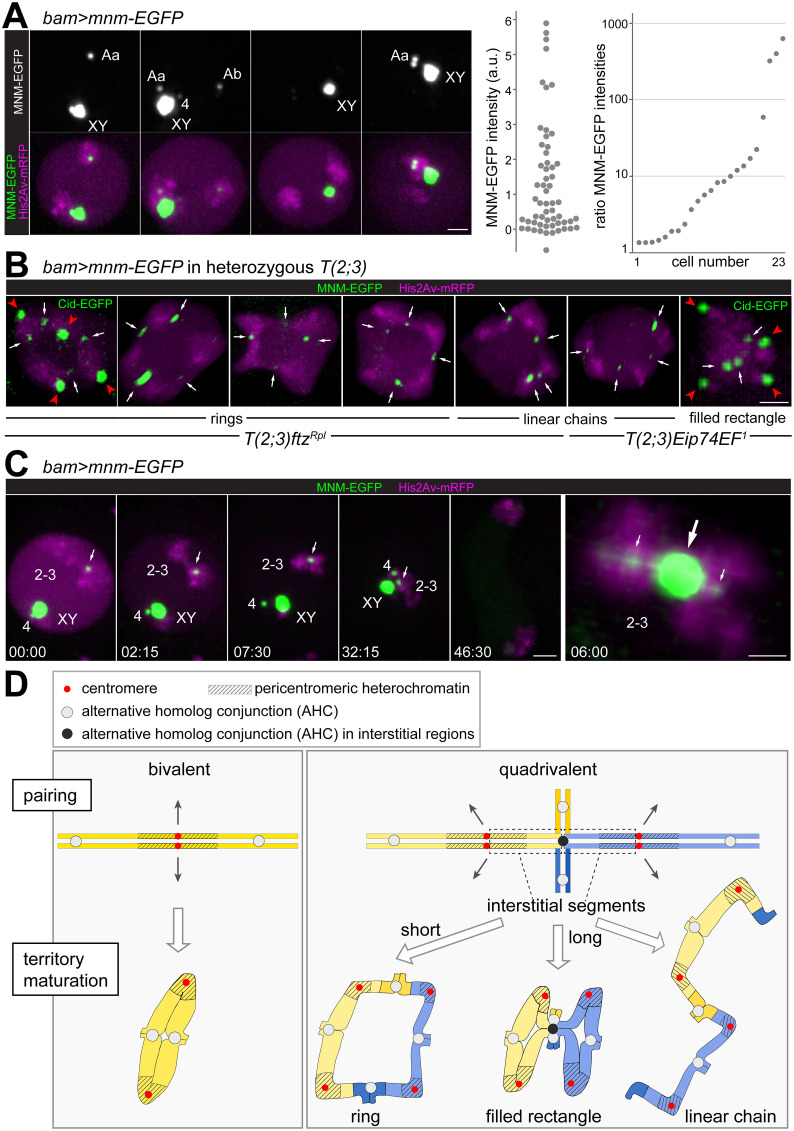
MNM-EGFP dots on quadrivalents. (**A-C**) AHC protein localization was analyzed by time-lapse imaging of spermatocytes with *bam*>*mnm-EGFP* and *His2Av-mRFP*. (**A**) Still frames from four distinct control spermatocytes at onset of NEBD I illustrate the variability in number and intensity of chromosomal MNM-EGFP dots. While the MNM-EGFP dot on the chrXY bivalent (XY) was invariably strong, autosomal dots on the bivalents of chr4 (4) and the two large autosomes (Aa and Ab) were detected as indicated. The swarm plot displays MNM-EGFP signal intensities associated with large autosomal bivalents (n = 58 from 29 spermatocytes). The dot plot presents the ratio of the MNM-EGFP signals associated with the two large autosomal bivalents (stronger/weaker signal) for all the 23 spermatocytes with signals above background on both large autosomal bivalents. (**B**) Quadrivalents from spermatocytes heterozygous for the indicated *T(2;3)* translocations. MNM-EGFP signals indicated by arrows. Quadrivalents displayed in the first and last panels are from spermatocytes co-expressing Cid-EGFP to mark centromeres (red arrowheads). (**C**) Still frames documenting progression through M I, as observed in unusual control spermatocytes, in which the two large autosomes (2–3) were conjoined by a strong MNM-EGFP dot (arrow, large arrow in last panel). The last panel displays the conjoined large autosomal bivalents at high magnification with enhanced green signals to reveal the weak MNM-EGFP dots within the conjoined bivalents (small arrows). Time (min:sec) relative to onset NEBD I. Scale bars = 4 μM (B, C and D left) and 1 μM (D right). (**D**) Model for the shaping of chromosomes before M I in *Drosophila* spermatocytes. Homologous chromosomes are paired during the S1 stage, followed by assembly of AHC protein complexes that maintain homolog conjunction during spermatocyte maturation. AHC is proposed to be intra-chromosomally restricted to around one spot per chromosome arm presumably at a random euchromatic location. After chromosome territory formation, the pericentromeric heterochromatin and the embedded centromeres of homologous chromosomes are moved apart during spermatocyte maturation, while AHC spots maintain homolog conjunction. In case of translocation heterozygotes, the length of the paired euchromatic segments and of the interstitial segments in the quadrivalent determines the probability of AHC complex assembly within these regions. The resulting distribution of AHC complexes determines the quadrivalent territory shape generated during spermatocyte maturation. See discussion for further explanation.

In principle, the ring shape displayed by quadrivalents in *T(2;3)ftz*^*Rpl*^ heterozygous spermatocytes might arise analogously as that of ring quadrivalents in case of reciprocal translocation heterozygotes during canonical meiosis. During the latter, the extended tight pairing of all the homologous regions on the normal and the structurally aberrant chromosomes produces a cross-shaped quadrivalent first (as shown schematically in Fig 6D). After CO formation in each limb of the cross (but not in the interstitial segments) and after SC disassembly, homologous regions are driven apart during diplotene except at the CO sites, resulting in the ring shape. Accordingly, in case of the ring quadrivalents formed during the achiasmate male meiosis in *Drosophila*, AHC proteins might be localized at positions equivalent to COs in canonical ring quadrivalents, as AHC is functionally replacing COs.

To evaluate this notion, we sought to analyze the localization of the AHC protein MNM in ring quadrivalents of *T(2;3)ftz*^*Rpl*^ heterozygous spermatocytes. In control spermatocytes without chromosomal aberrations, *bamP-GAL4-VP16* driven expression of *UASt-mnm-EGFP* allowed detection of the weak autosomal signals most effectively in comparison to the other known AHC proteins [5,16]. However, even with *bam*>*MNM-EGFP*, autosomal signals were not consistently detected on each large autosomal bivalent in every spermatocyte in the previous studies. Thus, before analyses of ring quadrivalents, we first assessed the detectability of autosomal MNM-EGFP signals by time-lapse imaging in control spermatocytes at the onset of NEBD I, a precisely defined developmental stage (Fig 6A). The intensity of MNM-EGFP signals associated with the large autosomal bivalents were quantified. All spermatocytes (n = 29, from five independent cysts) displayed MNM-EGFP dots above background on at least one of the two large autosomal bivalents. In 10% of the large autosomal bivalents, MNM-EGFP signals were not above background (Fig 6A, left graph) and those above background varied greatly in intensity even within a given spermatocyte (Fig 6A, right graph). The variability of autosomal MNM-EGFP dot intensities was far greater than that of centromeric Cid-EGFP dots [14] (S2 Fig). The autosomal MNM-EGFP dots were positioned in a medial region of the bivalents in between the homologous centromeres (S2 Fig). Counting the number of large autosomal MNM-EGFP dots was problematic because the lower end of their intensity range was at background levels. Considering exclusively the 40% of autosomal bivalents that displayed MNM-EGFP dots of comparable or higher intensity than centromeric Cid-EGFP dots, we never detected more than two of these strong MNM-EGFP dots per bivalent (n = 26 bivalents). Among the large autosomal bivalents with MNM-EGFP dots weaker than the centromeric Cid-EGFP dots, two (8%, n = 24) had three weak MNM-EGFP dots and all others fewer.

Overall, our analysis of MNM-EGFP dots in control spermatocytes was consistent with the suggestion that around one spot with AHC proteins per chromosome arm might maintain conjunction between large autosomal homologs in normal bivalents at the onset of M I.

The localization of MNM-EGFP on ring quadrivalents was analyzed analogously with heterozygous *T(2;3)ftz*^*Rpl*^ spermatocytes (Fig 6B). Apart from *bam*>*mnm-EGFP* and *His2Av-mRFP*, these spermatocytes also expressed *cid-EGFP* for some of the time-lapse imaging. Number and intensities of MNM-EGFP dots on the ring quadrivalents around NEBD I were variable, as in control bivalents. Importantly, the MNM-EGFP dots were positioned in between the centromeric Cid-EGFP dots in the medial region of the connecting chromatin (Fig 6B). Ring quadrivalents with one medial MNM-EGFP dot on each of the four chromatin connections were readily detected (Fig 6B and S14 Movie). Each cyst (n = 8) displayed one to three such quadrivalents. Thus, the MNM-EGFP dots on the ring quadrivalents were at positions corresponding to those of COs in ring quadrivalents during canonical meiosis.

In case of linear chain quadrivalents, which were present rarely in heterozygous *T(2;3)ftz*^*Rpl*^ spermatocytes and frequently in heterozygous *T(2;3)Eip74EF*^*1*^ spermatocytes, the MNM-EGFP dots were also localized in the medial region of the chromatin connections between pericentromeric heterochromatin blobs (Fig 6B and S15 Movie). In case of filled rectangle quadrivalents, which were observed in heterozygous *T(2;3)Eip74EF*^*1*^ spermatocytes beyond linear chains, MNM-EGFP dots were detected also in the central region (Fig 6B and S16 Movie). As explained in the discussion, we suggest that some of these centrally located MNM-EGFP dots might represent AHC protein spots within the interstitial segments of the quadrivalent, inhibiting the conversion of the quadrivalent into ring shape during territory maturation.

Overall, the observed medial localization of MNM-EGFP dots on the chromatin connecting the four centromeres in quadrivalents provided strong support for the suggestion that they are at sites of AHC.

Further support of the notion that MNM-EGFP dots indicate sites of AHC was obtained during the analysis of the control spermatocytes with *bam*>*mnm-EGFP* but without chromosomal aberrations. In 9% of these control spermatocytes (in 6 cells from three distinct cysts), the two large autosomal bivalents formed by chr2 and chr3, respectively, were abnormally conjoined into a quadrivalent with a single MNM-EGFP dot at the inter-bivalent conjunction site (Fig 6C). The two conjoined bivalents each displayed an additional intra-bivalent MNM-EGFP dot in their central regions, presumably mediating homolog conjunction (Fig 6C, last panel, and S17 Movie). The abnormally conjoined bivalents made prometaphase jumps in tight association, indicating robust physical linkage, which was lost during anaphase I in parallel with the disappearance of the chromosomal MNM-EGFP dots (Fig 6C and S18 Movie). In contrast, the large autosomal bivalents normally undergo the extensive jumps during prometaphase I independently [15]. The observed abnormal conjoining of the chr2-chr3 bivalents presumably resulted from MNM-EGFP overexpression, as this abnormality was never observed in a far greater number of cysts that we have analyzed previously by time-lapse imaging of spermatocytes without fluorescent AHC proteins [15] or with UNO-EGFP expressed in *uno* null mutants under control of the *uno cis*-regulatory region [5].

We add the caveat that MNM-EGFP dots might not necessarily mark exclusively AHC sites. This possibility was raised by the appearance of some of the linear chain quadrivalents observed in heterozygous *T(2;3)ftz*^*Rpl*^ and *T(2;3)Eip74EF*^*1*^ spermatocytes. During canonical meiosis, linear chain quadrivalents arise in reciprocal translocation heterozygotes when COs are established in all except one of the four limbs of the initial cross-shaped quadrivalent. Accordingly, with AHC spots instead of COs, linear chain quadrivalents formed during the achiasmate *Drosophila* male meiosis might be expected to have three AHC spots on the three connections in between the four centromeres. However, we observed linear chains displaying four MNM-EGFP dots, of which three were medial between pericentromeric heterochromatin blobs and a fourth in the terminal region on one end of the linear chain (Fig 6B and S15 Movie). The terminal MNM-EGFP dots raised the possibility that they might be on just one homolog instead on a site of homolog conjoining. To confirm the proposal that MNM-EGFP can be present during M I at chromosomal locations that are not conjunction sites, we analyzed MNM-EGFP dots in spermatocytes over-expressing the condensin II subunit Cap-H2. As recently demonstrated [11], Cap-H2 overexpression precludes the pairing of autosomes, resulting in random segregation of univalent autosomes during M I. Time lapse imaging clearly revealed the occasional presence of MNM-EGFP dots on these univalent autosomes during entry into M I (S3 Fig). We conclude that perdurance of MNM-EGFP foci until M I is not necessarily restricted to regions where two homologs are conjoined in close spatial association.

## Discussion

Shape and mechanical properties of chromosomes at M phase onset are crucial for their bi-orientation within division spindles and hence for accurate segregation of genetic information. Our analyses indicate that forces, which drive chromosomal entities apart, in combination with homolog conjunction determine the spatial organization of the bivalent chromosomes that are formed during prophase I of the achiasmate male meiosis in *D*. *melanogaster*.

A characteristic spatial separation of homologs all along the bivalents except at chiasmata was reported long ago for late prophase I of canonical meiosis, inspiring the term diplotene for this stage. Diplotene appearance of chromosomes arises after removal of the SC. A similar, partial separation of homologs also occurs during the achiasmate meiosis in *Drosophila* males. After an initial intimate pairing, homologs and even sister chromatids are driven apart during the stages S3 –S5 of *Drosophila* spermatogenesis [10], i.e., after formation during maturation of chromosome territories. It has been speculated that the forces separating homologs during both canonical meiosis and *Drosophila* male meiosis might be related [34]. *Drosophila* female meiosis, although canonical with regard to meiotic recombination and SC, does not include a classic diplotene stage. Instead, a dramatic compaction of all chromosomes into a spherical mass, the karyosome, occurs after pachytene in *Drosophila* oocytes. Direct mechanistic comparisons of homolog distancing during canonical diplotene and territory maturation in spermatocytes are therefore impossible in *Drosophila*.

The characteristic separation of homologs in canonical diplotene is often described as “repulsion”, as in Darlington’s comprehensive synthesis of early cytological research [35]. This term reflects initial suggestions that homolog separation might be driven by electrostatic repulsion. Recently, the Ki-67 protein, which coats chromosomes in mammalian cells at the start of mitosis, was shown to form repulsive molecular brushes [36]. Whether Ki-67 is involved in homolog repulsion during diplotene has not yet been reported. An obvious Ki-67 ortholog cannot be detected in *Drosophila*, precluding a straightforward evaluation of a potential involvement in chromosome dynamics in spermatocytes. Conceivably, functionally analogous proteins might be involved. However, instead of electrostatic repulsion, mechanical forces were also suggested to separate homologs in diplotene bivalents [37]. More recently, polymer models were shown to have remarkable explanatory power [38,39]. Strong radial repulsive forces are predicted to minimize overlap between the two homologs in meiotic bivalents, when chromosomes are modeled as bottlebrushes with polymer-like flexible chromatin loops attached to a central axis. Such chromosome models have gained considerable support by the demonstration that condensin complexes can extrude DNA loops in vitro [40,41]. Importantly, condensin II has been shown to be essential for chromosome territory formation in *Drosophila* spermatocytes [11,17]. In mutant spermatocytes lacking Cap-D3 or Cap-H2, the alpha-kleisin subunit of condensin II, chromocenter disruption fails and bivalents are not driven apart into distinct territories. However, live imaging has revealed that residual forces still act on the chromocenter in these mutant spermatocytes, attempting its disruption [11]. These currently unidentified forces might also achieve the extensive final separation of chromosome territories with substantial apparently DNA-free gaps in between, which cannot result exclusively from condensin-driven loop extrusion. Further work will be required to clarify the mechanism(s) of chromosome territory formation, as well as those responsible for the subsequent partial separation of homologs and sister chromatids during territory maturation. In a most simple scenario, territory formation and maturation might be driven at least in part by the same mechanisms. Over an extended phase, following the initial intimate homolog pairing, dispersive forces drive chromosomal entities apart during S2b until S5. Non-homologous associations of pericentromeric heterochromatin in the chromocenter are disrupted first [10,11], perhaps because they represent the weakest chromosomal interactions. Disruption of these non-homologous associations results in chromosome territories during the S2b stage. Inter-homolog and inter-sister associations are proposed to be stronger, explaining their delayed disruption during S3/4 [10,11]. Complete disruption of homologous associations is precluded by AHC [3,42], which is most likely applied, or rendered functional, just after territory formation [4,16].

Our present findings indicate that chromosomal entities are driven apart irrespective of their identity during territory formation. Previously, inhibition of autosomal homolog pairing by Cap-H2 overexpression was shown to result in formation of territories containing univalents instead of bivalents [11]. Here, a centromere-free chr3R fragment was shown to be separated away from the remainder of chr3 after acute fragment detachment by targeting Cas9 to the dodeca satellite repeat. In addition, an extra territory was also observed in spermatocytes when compound chromosomes replaced a normal large autosomal bivalent by two separate chromosomes containing the left and the right arms, respectively. Our analyses with *C(2L)* and *C(2R)*, which are thought to share partial heterochromatic homology around the centromeres, indicated that heterochromatic homology cannot prevent chromosomes from being moved apart during territory formation. On the other hand, euchromatic homology precludes chromosome separation during territory formation, even if it covers only a part of a chromosome, as indicated by our finding with spermatocytes heterozygous for reciprocal *T(2;3)* translocations, which form a single territory containing all large autosomal homologs. Interestingly, irrespective of the number of major territories (two in spermatocytes heterozygous for reciprocal *T(2;3)* translocations, three in wild-type and four in spermatocytes with compound chromosomes), the distribution of territories within the spermatocyte nucleus corresponded to that resulting from a maximization of the spatial separation between chromosomal entities. This pattern was most clearly revealed by our live imaging, which preserves native geometry in contrast to the widely used testis squash preparations.

While territory organization before the onset of M I does not appear to have been characterized previously in dipteran spermatocytes heterozygous for reciprocal translocations, cytological analyses have documented the presence of quadrivalents during achiasmate male M I in some dipterans (tsetse flies, onion flies) [43,44]. Beyond territory organization, we have also studied quadrivalent segregation during *Drosophila* male M I by time lapse imaging, because the quadrivalent models a partial failure of territory formation. Quadrivalents in *T(2;3)* heterozygotes correspond to mechanically coupled tandem bivalents, while two individualized chr2 and chr3 bivalents are present in normal M I. The four quadrivalent centromeres were found to segregate 2:2 in the majority of *T(2;3)* heterozygous spermatocytes, but 3:1 segregation was observed as well in up to 26% of the M I divisions, clearly emphasizing the importance of bivalent individualization by territory formation.

Importantly, our analyses revealed clear differences in quadrivalent shape and segregation pattern between the four distinct *T(2;3)* that we have analyzed. As discussed further below, these differences in quadrivalent shape provide important information concerning the spatial organization of AHC in chromosomes formed in preparation for MI. In case of *T(2;3)ftz*^*Rpl*^, the large majority of quadrivalents were ring-shaped and only few were in form of linear chains. In contrast, in *T(2;3)Eip74EF*^*1*^, the other simple type I translocation, quadrivalents were mostly linear chains and more rarely in form of rings or filled rectangles. In case of the complex type II translocations, *T(2;3)ap*^*Xa*^ and *T(2;3)TSTL*, linear chain quadrivalents were even more frequent. Moreover, instead of quadrivalents, the combination of a tri- and an univalent was observed with *T(2;3)ap*^*Xa*^, and the combination of two bivalents with *T(2;3)TSTL*, while with the simple translocations exclusively quadrivalents were formed.

The different forms of spatial chromosomal organization revealed by our time lapse imaging in spermatocytes heterozygous for *T(2;3)* translocations correspond to a striking extent to those reported during canonical meiosis long ago based on cytological analyses of primarily plant meiosis [29]. During canonical meiosis, maintenance of homolog pairing depends on COs. Therefore, the number and spatial distribution of COs are crucial for regular chromosome segregation during M I, necessitating careful regulation by processes including CO assurance, interference and homeostasis [45–47]. As a result, there is often around one CO per chromosome arm in many species. In *D*. *melanogaster* female meiosis, for example, there is an average of 1.2 COs per chromosome arm [48]. The number and distribution of COs is also crucial for the chromosomal organization of heterozygous reciprocal translocations during canonical meiosis. For example, when COs are absent within the interstitial segments but present on all of the four non-interstitial limbs of a cross-shaped quadrivalent generated by the initial intimate homolog pairing and synapsis, a ring quadrivalent is displayed during diplotene. When COs are present on only three limbs of the initial quadrivalent cross, but absent from one limb and from the interstitial segments, a linear chain quadrivalent results in diplotene. The length of the interstitial segments and of the limbs can vary between different translocation, and because length affects CO number, these parameters bias quadrivalent shape during diplotene. The various forms of chromosomal organization of heterozygous reciprocal *T(2;3)* translocations reported here during the achiasmate meiosis in *Drosophila* males are most readily explained by analogy with canonical meiosis. Among the three *T(2;3)* with known breakpoints, *T(2;3)ftz*^*Rpl*^, *T(2;3)Eip74EF*^*1*^ and *T(2;3)ap*^*Xa*^, the latter has one chromosome arm far shorter than all the others (Fig 3A–3C). This is proposed to result in a frequent failure to establish AHC on this short arm, and hence in the maximal frequency of linear chains observed for this *T(2;3)*. An additional failure to establish AHC on the other arm of this short-armed translocation chromosome would generate the combination of uni- and trivalent, which was indeed observed with this *T(2;3)*. Among the three *T(2;3)* with known breakpoints, *T(2;3)ftz*^*Rpl*^ has the shortest interstitial segments and hence particularly long limbs (Fig 3B). This is proposed (Fig 6D) to minimize the frequency of interstitial AHC and conversely maximize that of AHC on limbs, explaining the maximal frequency of ring-shaped quadrivalents detected for this *T(2;3)*. Conversely, the relatively long interstitial segments in *T(2;3)Eip74EF*^*1*^ (Fig 3A) are proposed to favor interstitial AHC and hence generate filled rectangles rather than ring quadrivalents (Fig 6D). Moreover, linear chains should be favored as well by the relatively long interstitial segments in *T(2;3)Eip74EF*^*1*^ (Fig 6D), because long interstitial segments are necessarily paralleled by short non-interstitial segments (Fig 3A). Overall, the various chromosomal forms of heterozygous reciprocal *T(2;3)* organization detected at the onset of M I demonstrate that AHC at M I onset is not present in every spermatocyte consistently along all the homologous euchromatic regions that are intimately paired initially [10]. Infallible AHC establishment along paired homologous segments is predicted to minimize differences in quadrivalent shape between spermatocytes with the same *T(2;3)* and also between spermatocytes with different *T(2;3)*s. However, profound and translocation-specific differences were exposed by our time-lapse imaging, suggesting that AHC establishment is probabilistic. As with COs during canonical meiosis, AHC establishment within a given arm may fail with a probability that is inversely correlated with arm length.

Direct detection of the chromosomal regions, where AHC maintains autosomal pairing, is technically demanding because autosomal signals obtained for the known AHC proteins cannot be detected consistently above background [3,5]. When detected, these signals are observed as foci on chromatin. In the *bam*>*mnm-EGFP* spermatocytes analyzed here, around 10% of the bivalents in controls failed to display an unambiguous MNM-EGFP dot during prometaphase I, but all these bivalents remained intact until anaphase I, indicating the presence of functional AHC. If AHC were absent in bivalents without MNM-EGFP signals above background, they should undergo premature separation into univalents, as observed in AHC null mutants during prometaphase I or already before [3,5,15]. Around 50% of the bivalents displayed two MNM-EGFP dots during prometaphase I. Most likely, therefore, there is around one AHC spot per chromosome arm. This remains a conjecture, because precise counting is impossible given the presence of bivalents lacking detectable MNM-EGFP dots and also because the two arm regions cannot be identified unequivocally with our imaging in case of normal bivalents. However, in ring quadrivalents of heterozygous *T(2;3)ftz*^*Rpl*^ spermatocytes, arm regions were separated widely apart. Ring quadrivalents with one MNM-EGFP dot per arm region were readily detected, consistent with the proposal of around one AHC spot per arm. Moreover, the intra-chromosomal positions of the MNM-EGFP dots in bivalents and quadrivalents indicated that they are localized at functional AHC sites. The great majority of the chromosomal MNM-EGFP dots around NEBD I were at the positions of homolog conjunction, i.e., medial in between bi-oriented homologous centromeres. In addition, the occasional ectopic conjoining of the large autosomal bivalents observed in *bam*>*mnm-EGFP* spermatocytes was accompanied by a MNM-EGFP dot in between the conjoined bivalents. Overall, our results argue strongly for the presence of around one AHC spot per chromosome arm, comparable to the number of COs during canonical meiosis.

The suggestion that autosomal AHC might be restricted to about one spot per chromosome arm raises important questions. Which mechanisms control their number and chromosomal location? In principle, the presence of unique DNA sequences for AHC protein recruitment at one specific locus per chromosome arm might solve the problem. This mode is used in *Drosophila* spermatocytes for the conjunction of sex chromosomes [8]. These chromosomes lack euchromatic homology, but they both contain rDNA repeats in the centromere proximal heterochromatin. These rDNA repeats mediate chrXY conjunction by recruiting the AHC proteins. However, efforts to identify analogous unique and defined AHC sites in autosomes have failed and argued for the notion that a particular AHC site might be selected among many potential locations independently in each spermatocyte and bivalent [7,8]. Thus, the positioning of AHC site might be analogous to that of COs, which are derived from meiotic double-strand breaks (DSBs). In *D*. *melanogaster* oocytes, meiotic DSBs can occur almost everywhere within euchromatin with no indication for hot spots, while they are rare or absent in heterochromatin [48]. After formation of DSBs, some are chosen from a 2–3 fold excess and designated for repair into COs, while the remainder is repaired via non-crossover (NCO) pathways. Interference acts during CO/NCO differentiation, regulating CO distribution towards 1.2 per chromosome arm. As in case of COs, we do not observe MNM-EGFP dots within centromere-proximal heterochromatin in large autosomes. The strong variability of the autosomal MNM-EGFP dot intensities might reflect differences in DNA sequences and/or chromatin structure at the particular location chosen as AHC site. This strong variability in MNM-EGFP dot intensity might also point to a mechanism that keeps the number of AHC sites per chromosome within bounds. Autosomal territories at early stages appear to be characterized by a higher number of MNM-EGFP dots compared to mature bivalents at NEBD I. We speculate that MNM-EGFP, which includes intrinsically disordered regions, might form phase-separated bodies and that these might participate in an Ostwald ripening-type coarsening process favoring AHC protein transfer from smaller to larger condensates [49]. Similar models have been implicated in CO interference recently in *C*. *elegans* [50–52], although with a prominent role of the SC, which is absent in *Drosophila* spermatocytes.

Our findings prompt us to propose a working model for chromosome shaping before M I in *Drosophila* spermatocytes (Fig 6D). Two rules appear to guide this process. The intrachromosomal localization of AHC in case of the large autosomes is proposed to correspond to that of COs in canonical meiosis (rule 1). Like COs, AHC is not established within pericentromeric heterochromatin. Moreover, rather than extending over all or most of the homologous euchromatic arm regions, AHC seems to be strongly confined to a spot. The number of AHC spots per euchromatic arm appears to be just around one, and the intrachromosomal location might vary between different spermatocytes. The forces that drive the formation and maturation of chromosome territories attempt to achieve maximal spatial separation between chromosomal entities (rule 2). The resulting spatial distancing between homologs seems equivalent to “repulsion” during diplotene of canonical meiosis. AHC spots preclude the spatial separation of the conjoined chromosomal entities locally. In contrast, the pericentromeric heterochromatin regions with the embedded centromeres are moved apart widely as they are free of AHC. In case of a quadrivalent, if each of the four non-interstitial segments has an AHC spot but not the interstitial segments, a ring-shaped quadrivalent will be generated with the four homologous centromeres maximally apart (Fig 6D). A linear chain quadrivalent will result, if one of the four non-interstitial segments lacks an AHC spot (Fig 6D). Moreover, if AHC spots are present not just within the non-interstitial segments but also within interstitial segments, a quadrivalent shape similar to a filled rectangle will result (Fig 6D).

While this model emphasizes parallels between homolog linkage by AHC during the achiasmate male meiosis of *D*. *melanogaster* and by COs during canonical meiosis, as previously proposed [8], further work is clearly needed to clarify the extent of similarity. In the light of evolution, it should not come as a surprise if the invention of AHC was achieved largely by modification of canonical processes.

## Materials and methods

### *Drosophila* lines

The lines with the following mutations or transgenes have been described before: *UAS-Cas9* (II) (Bloomington *Drosophila* stock Center (BDSC) # 58985), *UAS-Cas9* (III) (BDSC # 58986), C(3L)*, st; C(3R)*, *e[s]* (BDSC # 1624), *C(2L)RM-P1*, *b; C(2R)RM-P4*, *px* (BDSC # 713), *C(2)EN*, *b pr* (BDSC # 1112), *T(2;3)ap[Xa]*, *ap[Xa]/ TM6B*, *Tb* (BDSC # 1788), w[*]; P{w[+mC] = UAS-Katushka.*I}2/ T(2;3)TSTL*, *CyO*: *TM6B*, *Tb* (BDSC # 56504), *T(2;3)ftz[Rpl]*, *ftz[Rpl]/ TM3*, *ry[RK] Sb Ser* (BDSC # 2219), *T(2;3)Eip74EF*^*1*^, *Eip74EF e/ TM6B*, *Tb* (BDSC # 29973), *His2Av-mRFP* and *g-cid-EGFP-cid* [53], *UASt-mnm-EGFP II*.*2* [16], *UASt-EGFP-Cap-H2* [11], *bamP-GAL4-VP16* [54].

The *UAS-gRNA_dodeca* and *UAS-gRNA_acedod* transgenic lines were generated essentially as described [55] with the pCaSpeR4 derived plasmids described further below. The constructs were injected into *w*^1118^ embryos (BestGene Inc., Chino Hills, CA, USA).

Standard crossing was used for the generation of the various strains used for experimental analyses. The genotypes of the flies analyzed are described in detail in the supporting information (S1 Table). All flies analyzed were raised at 25°C.

### Plasmids

For the generation of pCaSpeR4-gRNA-dodeca and pCaSpeR4-gRNA-acedod we first annealed oligonucleotides including the desired gRNA sequence (see below). For gRNA_dodeca, LV042 and LV043 were annealed, for gRNA_acedod, LV044 and LV045. The sequences of the oligonucleotides were (5’– 3’): TGCAGGACCAGTACGGGACCAGTA (LV042), AAACTACTGGTCCCGTACTGGTCC (LV043), TGCACCTGGTCATGCCCTGGTCAT (LV044), and AAACATGACCAGGGCATGACCAGG (LV045). The annealed oligo were inserted into pCFD6 (Port and Bullock, 2016) (Addgene, #73915) after digesting this vector with BbsI. A BamHI fragment was excised from the resulting pCFD6 derivatives and inserted into the corresponding restriction site of pCaSpeR4.

The CRISPR/cas9 target online predictor CCTop [56] was used for the prediction of target sites recognized by gRNA_dodeca (5’-GGACCAGTACGGGACCAGTA-3’) and gRNA_acedod (5’-CCTGGTCATGCCCTGGTCAT-3’). Allowing for up to 4 mismatches, a total of 22394 target sites were detected in the *Drosophila* genome in case of gRNA_dodeca: 1 intergenic site on chrX (with 4 mismatches), 1 site within *CG13384* on chr2L (with 4 mismatches), 8 sites clustered about 29 kb upstream of *haspin* on chr2R (with 3–4 mismatches), 8223 sites on chr3R within the first 0.5 MB of the reference genome (release 6), which do not include any annotated genes. 2926 of these pericentromeric sites on chr3R displayed a perfect match. 12 additional sites were detected (3 perfect match and the remaining 9 with 3–4 mismatches) within an intron of *Myo81F/CG45784*, which is the most centromere-proximal gene on chr3R. Yet another site (with 4 mismatches) on chr3R was intergenic near *CG56154*. The remaining 14148 sites (5859 with a perfect match) were on unannotated contigs. In contrast, only 1 site with 4 mismatches was found with the inverted complement gRNA_acedod (5’-CCTGGTCATGCCCTGGTCAT-3’) on chr2R.

### Fertility tests

For analysis of male fertility, we crossed single males with three *w* virgin females. Ten replicate crosses were started. After two days of mating, crosses were transferred into a fresh vial. After an additional two days, all adult flies were discarded, followed by counting all of the adult progeny that developed subsequently at 25°C.

### Fixation and labeling of testis preparations

Testis squash preparations were made and stained essentially as described previously [57], according to protocol 3.3.2, except that a distinct mounting medium (70% glycerol, 1% n-propyl gallate, 0.05% p-phenylenediamine, 50 mM Tris-HCl pH 8.5) was used. For immunolabeling, mouse monoclonal anti-Lamin Dm0 antibody ADL67.10 (Developmental Studies Hybridoma Bank) (1:50) and Alexa568-conjugated goat antibody against mouse IgG (Invitrogen, A11004) (1:500) were used. For DNA staining, testes were incubated for 10 minutes in PBS, 0.1% Triton X-100 (PBTx) containing Hoechst 33258 (1 μg/ml). After three washes with PBS, a drop of mounting medium was applied on the slide before adding a cover slip. Generally, about 20 dissected testes were mounted per slide. Images (z stacks) were acquired using a Zeiss Cell Observer HS wide-field microscope using 40×/0.75 or 63×/1.4 objectives.

### Testis preparations for live imaging

Time-lapse imaging of progression through meiosis was performed as recently described [15]. In brief, testes from pupal or young adult males were dissected in Schneider’s *Drosophila* Medium (Invitrogen, #21720) supplemented with 10% fetal bovine serum (Invitrogen) and 1% penicillin/streptomycin (Invitrogen, #15140). The dissected testes were transferred into 45 μl of medium in a 35 mm glass bottom dish (MatTek Corporation, #P35G-1.5-14-C) and opened with fine tungsten needles to release the cysts. To reduce sample movements, 15 μl of 1% w/v methylcellulose (Sigma, #M0387) was added. A wet filter paper was placed inside along the dish wall before sealing the lid with parafilm. For long-term time-lapse imaging of territory formation over up to six hours, 1.5 ml of medium and 0.5 ml of methylcellulose were used. No wetted filter papers were used in these experiments. Imaging was performed at 25°C in a room with temperature control using a spinning disc confocal microscope (VisiScope with a Yokogawa CSU-X1 unit combined with an Olympus IX83 inverted stand and a Photometrics evolve EM 512 EMCCD camera, equipped for red/green dual channel fluorescence observation; Visitron systems, Puchheim, Germany). A 60×/1.42 oil immersion objective was used for acquisition of z stacks. The z stacks acquired for analysis of progression through M I comprised 46 focal planes spaced by 500 nm. The stacks were acquired at intervals of 45 seconds or around 10 seconds for accurate centromere tracking to resolve the process of *T(2;3)* quadrivalent orientation within the spindle.

### Image processing and analysis

The IMARIS software (Bitplane; versions 8.4.0, 9.2.0, 9.7.2) was used for spot detection in the channel with the Cid-EGFP signals, setting the parameter “estimated xy diameter” to 500 nm with background subtraction as described [15]. For the analysis of chromosome segregation during M I, centromeric Cid-EGFP spots were tracked using the algorithm “Autoregressive Motion” of IMARIS software. The resulting tracks were corrected as follows. Spots for centromeric signals that were not recognized automatically were added manually. Conversely, spots assigned to background signals were deleted. Manual correction of tracks was readily possible because the distinct features of the His2Av-mRFP signals associated with centromeric dot signals were also taken into account during visual correction, while His2Av-mRFP signals were ignored during the automatic detection by the IMARIS software. Scoring of NEBD and later phase transitions during progression through M I was done as described [15]. The distance separating homologous centromere was measured in 3D as described [15]. The volume of the tetrahedron defined by the four centrosomes of a *T(2;3)* quadrivalent was calculated based on the spatial coordinates (x, y, z) of the Cid-EGFP spots identified by IMARIS software from the time points two minutes after NEBD I. For special emphasis of chromosome territories in some images and movies, isosurfaces were generated after live imaging in the His2Av-mRFP channel using IMARIS software. Centromeric signals observed during progression through M I were assigned to specific chromosomes using criteria as previously described [15]. The bivalents formed by the large autosomes (chr2 and chr3) in control spermatocytes are characterized by a relatively abundant mass of associated His2Av-mRFP that is symmetric along the inter-centromere axis, while it is asymmetric in case of the sex chromosome bivalent [15]. The bivalent of chr2 and that of chr3 can be distinguished during early prometaphase I, because the latter as more pronounced His2Av-mRFP-positive heterochromatin blobs adjacent to the centromeres during early prometaphase [15]. Accordingly, we have also assigned chr2 and chr3 centromeres, respectively, in *T(2;3)* quadrivalents. Because the differences in centromere-proximal His2Av-mRFP-positive heterochromatin blobs displayed during early prometaphase I are subtle, our assignment of chr2 and chr3 centromeres in quadrivalents might not be free of mistakes, which however do not affect our conclusion that the four centromeres of the *T(2;3)ftz*^*Rpl*^ ring quadrivalent can segregate 2:2 in three distinct modes (adjacent-1, adjacent-2 and alternate). Potential mistakes in the assignment of chr2 and chr3 centromeres in quadrivalents only affect the distinction of a particular adjacent 2:2 segregation as either adjacent-1 or adjacent-2.

MNM-EGFP signal intensities associated with large autosomal bivalents in spermatocytes with *bam*>*mnm-EGFP* and *His2Av-mRFP* were quantified at the time point revealing the start of NEBD I. Maximum intensity projections were exported as tif files and analyzed using Image J. Regions of interest (ROIs) containing a large autosomal bivalent were drawn manually based on the His2Av-mEFP signals, followed by determination of the integrated pixel intensities in the green channel within these ROIs. For background correction, each ROIs was dilated by 15 pixels. The region present exclusively in the dilated larger ROI but not in the initial small ROI was considered to contain exclusively background signals in the green channel. The mean pixel intensity within this local background region was multiplied by the area of the small ROI and subtracted from the small ROI’s integrated pixel intensities in the green channel.

Figures display maximum intensity projections unless stated otherwise. These projections were generated using ImageJ for wide-field images and IMARIS for confocal images. Export of projections from IMARIS as movies or still frames after live imaging was made with interpolated image display. Moreover, display parameters for the His2Av-mRFP signals were adjusted manually over time to reveal chromosomes clearly throughout the time course, thereby correcting photobleaching and partially also the changes in the extent of chromosome condensation during M I. Graphs were generated with Microsoft Excel or GraphPad Prism. P values were calculated using a two-tailed student t-test (* = p < 0.05; ** = p < 0.01; *** = p < 0.001). Adobe Photoshop, Adobe Illustrator and Inkscape were used for production of figures.

## Supporting information

S1 Text Chr3 segregation defects during meiotic divisions after dodeca satellite targeting in early spermatocytes. (PDF)

S1 Fig Quadrivalent distortion during centromere reorientation and attachment failure of sex chromosome bivalent in *T(2;3)Eip74EF*^*1*^ heterozygous spermatocyte. Still frames after time lapse imaging of His2Av-mRFP and Cenp-A/Cid-EGFP in spermatocytes heterozygous for *T(2;3)Eip74EF*^*1*^ illustrate progression through M I until anaphase onset. Time (min:sec) relative to onset NEBD I. Territories formed by the quadrivalent [T(2;3)] and by the other chromosomes (XY4) are indicated in the first frame, and equatorial plane (dashed lines) in the last two frames. Tracked centromeres are marked by spheres with colors indicating the associated chrX and chrY (red), chr4 (green), chr2 and chr3 (yellow and blue). Re-orientation of a quadrivalent centromere (dark blue sphere) occurs between 10:00 and 10:56 during prometaphase I. Centromeres of the sex chromosome bivalent (arrowheads in the last two frames) fail to attach to the distant spindle pole on the other side of the quadrivalent. Scale bar = 3 μm.
(PDF)

S2 Fig Intensity and position of autosomal MNM-EGFP dots relative to centromeres. Control spermatocytes expressing *His2Av-mRFP*, *cid-EGFP* and *bam*>*mnm-EGFP* were analyzed by time-lapse imaging. The same still frame from early prometaphase I is displayed three times with increasing enhancement of green signal intensities from left to right (low, medium and high). The chrXY bivalent (XY) and the large autosomal bivalents (Aa and Ab) are indicated, as well as visible green dots representing MNM-EGFP (arrows) and centromeric Cid-EGFP (red arrowheads). MNM-EGFP dots and centromeric Cid-EGFP dots on large autosomal bivalents could be differentiated because the former but not the latter disappeared during exit from M I. Centromeric Cid-EGFP dots have comparable intensities in contrast to the highly variable MNM-EGFP dots localized at a medial position between the centromeres. The strong MNM-EGFP dot on the chrXY bivalent presumably masks Cid-EGFP signals on the bivalents with chrXY and chr4, as well as MNM-EGFP signals on the chr4 bivalent. Scale bar = 2 μM.
(PDF)

S3 Fig Autosomal MNM-EGFP dots on univalents. Progression through M I was analyzed by time-lapse imaging of spermatocytes with *His2Av-mRFP* and *bamP-GAL4-VP16* driving expression of either only *UASt-EGFP-Cap-H2* (top row) or both *UASt-EGFP-Cap-H2* and *UASt-mnm-EGFP* (middle and bottom rows). *bam*> *EGFP-Cap-H2* precludes the pairing of autosomal homologs, which normally occurs in early spermatocytes. Protein instability of EGFP-Cap-H2 after transient *bamP-GAL4-VP16* driven expression in early spermatocytes results in an absence of EGFP-Cap-H2 signals during M I (top row). In contrast, MNM-EGFP dots (middle and bottom row) perdure until M I. Beyond the strong MNM-EGFP dots on the chrXY bivalent (XY), autosomal univalents displayed weaker signals (arrows) of variable intensities (arrow size) comparable to control spermatocytes (Fig 6A). Still frames from spermatocytes progressing through M I are displayed in the top and middle rows. Time (min:sec) relative to onset NEBD I. The bottom row displays additional examples of prometaphase I spermatocytes. Scale bars = 2 μM.
(PDF)

S1 Movie Chromosome territories in control spermatocyte. A single spermatocyte during late S5 is displayed after time-lapse imaging of a spermatocyte cyst expressing Cenp-A/Cid-EGFP (green) and His2Av-mRFP (magenta). After a first full rotation, a second rotation is presented with colored isosurfaces around the chromosome territories (chrXY4 in red, chr2 in yellow, chr3 in blue). A Gaussian filter was applied to the His2Av-mRFP channel before isosurface generation. The same spermatocyte is shown in Fig 2D in a maximum intensity projection.
(MP4)

S2 Movie Chromosome territories after Cas9-mediated dodeca satellite cutting in spermatocytes. Cas9 and the gRNA_dodeca targeting the dodeca satellite repeat were expressed in spermatocytes along with Cenp-A/Cid-EGFP (green) and His2Av-mRFP (magenta) for time-lapse imaging. A single spermatocyte during late S5 is displayed. After a first full rotation, a second rotation is presented with colored isosurfaces around the chromosome territories (chrXY4 in red, chr2 in yellow, chr3 in blue). A Gaussian filter was applied to the His2Av-mRFP channel before isosurface generation. The same spermatocyte is shown in Fig 2D in a maximum intensity projection.
(MP4)

S3 Movie Progression through M I after Cas9-mediated dodeca satellite cutting. Cas9 and a gRNA targeting the dodeca satellite repeat were expressed in spermatocytes along with Cenp-A/Cid-EGFP (green) and His2Av-mRFP (magenta) for time-lapse imaging. Progression through M I, as observed in the spermatocyte presented in S1A Fig, is shown. To illustrate chromosome territories, the first time point (7:30 min before onset NEBD I) is rotated first without and thereafter with colored isosurfaces around the territories (chrXY4 in red, chr2 in yellow, chr3 in blue). A Gaussian filter was applied to the His2Av-mRFP channel before isosurface generation. Subsequent progression until anaphase I is shown as a maximum intensity projection with tracks indicating the position of the chr3 centromeres. Time (min:sec) is indicated.
(MP4)

S4 Movie Quadrivalent chromosome territory in *T(2;3)ftz*^*Rpl*^ heterozygous spermatocyte during S5. Cenp-A/Cid-EGFP (green) and His2Av-mRFP (magenta) were used for visualization of chromosome territory organization by live imaging. The quadrivalent territory in a ring configuration presented in Fig 4C is shown with rotations with and without an isosurface around territory.
(MP4)

S5 Movie Ring quadrivalent in *T(2;3)ftz*^*Rpl*^ heterozygous spermatocyte during S6. Cenp-A/Cid-EGFP (green) and His2Av-mRFP (magenta) were used for visualization of chromosome territory organization by live imaging. Two S6 spermatocytes with partially condensed, ring-shaped quadrivalent territories, including the one presented in Fig 4C, are shown with partial rotations.
(MP4)

S6 Movie Linear chain quadrivalent in *T(2;3)ftz*^*Rpl*^ heterozygous spermatocyte during S6. Cenp-A/Cid-EGFP (green) and His2Av-mRFP (magenta) were used for visualization of chromosome territory organization by live imaging. The S6 spermatocyte presented in Fig 4C with a partially condensed quadrivalent territory in a linear chain configuration is shown with a rotation.
(MP4)

S7 Movie Quadrivalent chromosome territory in *T(2;3)Eip*^*1*^ *heterozygous* spermatocyte during S5. Cenp-A/Cid-EGFP (green) and His2Av-mRFP (magenta) were used for visualization of chromosome territory organization by live imaging. The quadrivalent territory presented in Fig 4C is shown with rotations with and without an isosurface around territory.
(MP4)

S8 Movie *T(2;3)ftz*^*Rpl*^ heterozygous spermatocyte with 3:1 segregation of ring quadrivalent during M I. Cenp-A/Cid-EGFP (green) and His2Av-mRFP (magenta) were used for analysis of progression through M I in spermatocytes heterozygous for *T(2;3)ftz*^*Rpl*^. The spermatocyte presented in Fig 5A is shown with tracked centromeres marked by colored spheres: chrX and chrY (red), chr4 (green), chr2 and chr3 (yellow and blue).
(MP4)

S9 Movie *T(2;3)ftz*^*Rpl*^ heterozygous spermatocyte with 2:2 segregation of ring quadrivalent during M I. Cenp-A/Cid-EGFP (green) and His2Av-mRFP (magenta) were used for analysis of progression through M I in spermatocytes heterozygous for *T(2;3)ftz*^*Rpl*^. The spermatocyte presented in Fig 5C is shown with tracked centromeres marked by colored spheres: chrX and chrY (red), chr4 (green), chr2 and chr3 (yellow and blue).
(MP4)

S10 Movie Ring quadrivalent at metaphase I with adjacent 2:2 orientation of centromeres. Spermatocytes heterozygous for *T(2;3)ftz*^*Rpl*^ were analyzed by time lapse imaging using Cenp-A/Cid-EGFP (green) and His2Av-mRFP (grey). The spatial organization of the quadrivalent presented in Fig 5D is highlighted with an isosurface and a rotation around the spindle axis. Centromeres were marked using yellow and blue spheres for chr2 and chr3, respectively.
(MP4)

S11 Movie Shape transformation of ring quadrivalent accompanying alternate 2:2 orientation of centromeres. Spermatocytes heterozygous for *T(2;3)ftz*^*Rpl*^ were analyzed by time lapse imaging using Cenp-A/Cid-EGFP (green) and His2Av-mRFP (grey). The spatial transformation of the four quadrivalent centromeres from a ring to an anti-parallelogram configuration presented in Fig 5E is documented with the five consecutive time points that end with the final alternate 2:2 orientation of the centromeres. Centromeres were marked using yellow and blue spheres for chr2 and chr3, respectively.
(MP4)

S12 Movie Ring quadrivalent at metaphase I with alternate 2:2 orientation of centromeres. Spermatocytes heterozygous for *T(2;3)ftz*^*Rpl*^ were analyzed by time lapse imaging using Cenp-A/Cid-EGFP (green) and His2Av-mRFP (grey). The spatial organization of the quadrivalent presented in Fig 5F is highlighted with an isosurface and a rotation around the spindle axis. Centromeres were marked using yellow and blue spheres for chr2 and chr3, respectively.
(MP4)

S13 Movie Quadrivalent distortion during centromere reorientation and attachment failure of sex chromosome bivalent in *T(2;3)Eip74EF*^*1*^ heterozygous spermatocyte during M I. Cenp-A/Cid-EGFP (green) and His2Av-mRFP (magenta) were used for the analysis of condensation and segregation of chromosomes during M I in spermatocytes heterozygous for *T(2;3)Eip*^*1*^. The spermatocyte presented in S1 Fig is shown with rotations at time points before and during the final chromosome condensation and with tracked centromeres marked by colored spheres: chrX and chrY (red), chr4 (green), chr2 and chr3 (yellow and blue).
(MP4)

S14 Movie MNM-EGFP dots on ring quadrivalent. Spermatocyte heterozygous for *T(2;3)ftz*^*Rpl*^ with *bam*>*mnm-EGFP* (green) and *His2Av-mRFP* (magenta) displayed in Fig 6B (fourth panel from left). The complete cell is shown during a first 360° rotation and exclusively the quadrivalent region during a second 360°C rotation.
(MP4)

S15 Movie MNM-EGFP dots on linear chain quadrivalent. Spermatocyte heterozygous for *T(2;3)ftz*^*Rpl*^ with *bam*>*mnm-EGFP* (green) and *His2Av-mRFP* (magenta) displayed in Fig 6B (fifth panel from left). The complete cell is shown during a first 360° rotation and exclusively the quadrivalent region during a second 360°C rotation.
(MP4)

S16 Movie MNM-EGFP dots on filled rectangle quadrivalent. Spermatocyte heterozygous for *T(2;3)Eip74EF*^*1*^ with *bam*>*mnm-EGFP* (green), *Cenp-A/cid-EGFP* (green) and *His2Av-mRFP* (magenta) displayed in Fig 6B (seventh panel from left). The complete cell is shown during a first 360° rotation and exclusively the quadrivalent region during a second 360°C rotation. Centromeric Cid-EGFP dots and MNM-EGFP dots associated with the quadrivalent are marked with red and white spheres, respectively. The additional green signals associated with chrXY and chr4 bivalent are not labeled.
(MP4)

S17 Movie MNM-EGFP dots in conjoined large autosomal bivalents. Conjoined chr2 and chr3 bivalents from a spermatocyte with *bam*>*mnm-EGFP* (green) and *His2Av-mRFP* (magenta) displayed in Fig 6C (last panel from left).
(MP4)

S18 Movie Progression through M I of a spermatocyte with conjoined chr2 and chr3 bivalents. Progression through M I of the spermatocyte with *bam*>*mnm-EGFP* (green) and *His2Av-mRFP* (magenta) that is also presented in Fig 6C. The image stack sequence is shown twice. During the repetition, display settings in the green channel were adjusted to reveal the relatively weak MNM-EGFP dots within the conjoined bivalents. These display settings saturate the signals of the stronger MNM-EGFP dot in between the two conjoined bivalents. This saturation is minimized by the display settings during the first sequence. Time (hours:min:sec) is indicated.
(MP4)

S1 Table Description of the analyzed genotypes. (XLSX)

S2 Table Source data. (XLSX)
