# Supplementary material for: Dispersive forces and resisting spot welds by alternative homolog conjunction govern chromosome shape in Drosophila spermatocytes during prophase I
Source: PLoS Genet. 2022 Jul 27;18(7):e1010327. doi: 10.1371/journal.pgen.1010327 (PMC9359577; doi:10.1371/journal.pgen.1010327)
Supplement: S1 Text — (PDF) [file pgen.1010327.s001.pdf]

S1 Text. Defective chr3 segregation during meiotic divisions after dodeca satellite targeting in early spermatocytes

To study progression through M I after Cas9-mediated dodeca satellite cleavage, we analyzed those 28 spermatocytes that remained completely within the z stacks acquired by time-lapse imaging of four independently prepared and imaged cysts. Only one of these 28 spermatocytes displayed an apparently wild-type phenotype. In the remaining 27 spermatocytes, the chr3 bivalent displayed abnormalities, in contrast to the sex chromosome bivalent and the autosomal bivalents of chr2 and chr4.

The time-lapse analysis demonstrated that during M I, the centromeres of chr3 retained a connection with the main chromatin masses of chr3 rather than being completely released from chr3 by dodeca satellite targeting. This observation is consistent with the recent finding [1] that the dodeca satellite repeats are located on the right side of the centromere of chr3 rather than surrounding this centromere as originally proposed [2]. In only one exceptional cell (Fig D), one centromeric Cid-EGFP dot made independent prometaphase I jumps more than 3.5 µm away from a chromatin mass with the second chr3-associated Cid-EGFP dot. However, during exit from M I even this apparently detached centromere displayed a behavior, which clearly exposed a remaining connection to chr3 chromatin.

Time-lapse analysis of progression through M I also exposed residual interconnections between the two chr3 sub-territories apparent during spermatocyte maturation after dodeca satellite targeting in early spermatocytes. Around NEBD I, the two chr3 sub-territories were pulled together by chromosome condensation and the split appearance with two independent sub-territories was converted into a bi-lobed chromosome mass that displayed concerted movements during prometaphase I (Fig A and S3 Movie).

Integration of the abnormal chr3 bivalent into the M I spindle was variable. The two closely spaced centromeres were either segregated to the same pole after syntelic attachment (Fig A and S3 Movie) or segregated apart after bi-orientation (Fig B). In cells displaying only a single Cid-EGFP dot on the chr3 bivalent at NEBD I, chromosome condensation or interactions with spindle microtubules resulted in splitting of the centromere dot at least transiently. Anaphase I in spermatocytes with bi-oriented chr3 centromeres ensued with chromosome bridges (Fig B), leading to the formation of 1-3 micronuclei at the onset of interkinesis in six of the 28 analyzed M I divisions (Fig C). Testis squash preparations provided further confirmation that dodeca satellite targeting in early spermatocytes caused meiotic chromosome segregation defects, as early spermatid nuclei had a highly variable DNA content (Fig E).

Based on our observations we propose that the targeting of Cas9 to the dodeca satellite caused double-strand DNA breaks (DSBs) between the centromere and the right arm of chr3 (chr3R). As *bamP-GAL4-VP16* induces just a transient pulse of *UAS* target gene expression in early spermatocytes [3], reconnection of chr3R appears to prevail eventually over complete detachment. Given the high number of dodeca satellite repeats, many cycles of separation and reconnection might occur while cutting activity is high in early spermatocytes. As four chr3 chromatids are kept in close association by sister cohesion and homolog pairing during the stages of cutting, DSB repair is likely to produce complex interlinkage of fragments and chromatids. Such interconnections presumably preclude the separation of homologous centromeres that normally occurs during spermatocyte maturation.

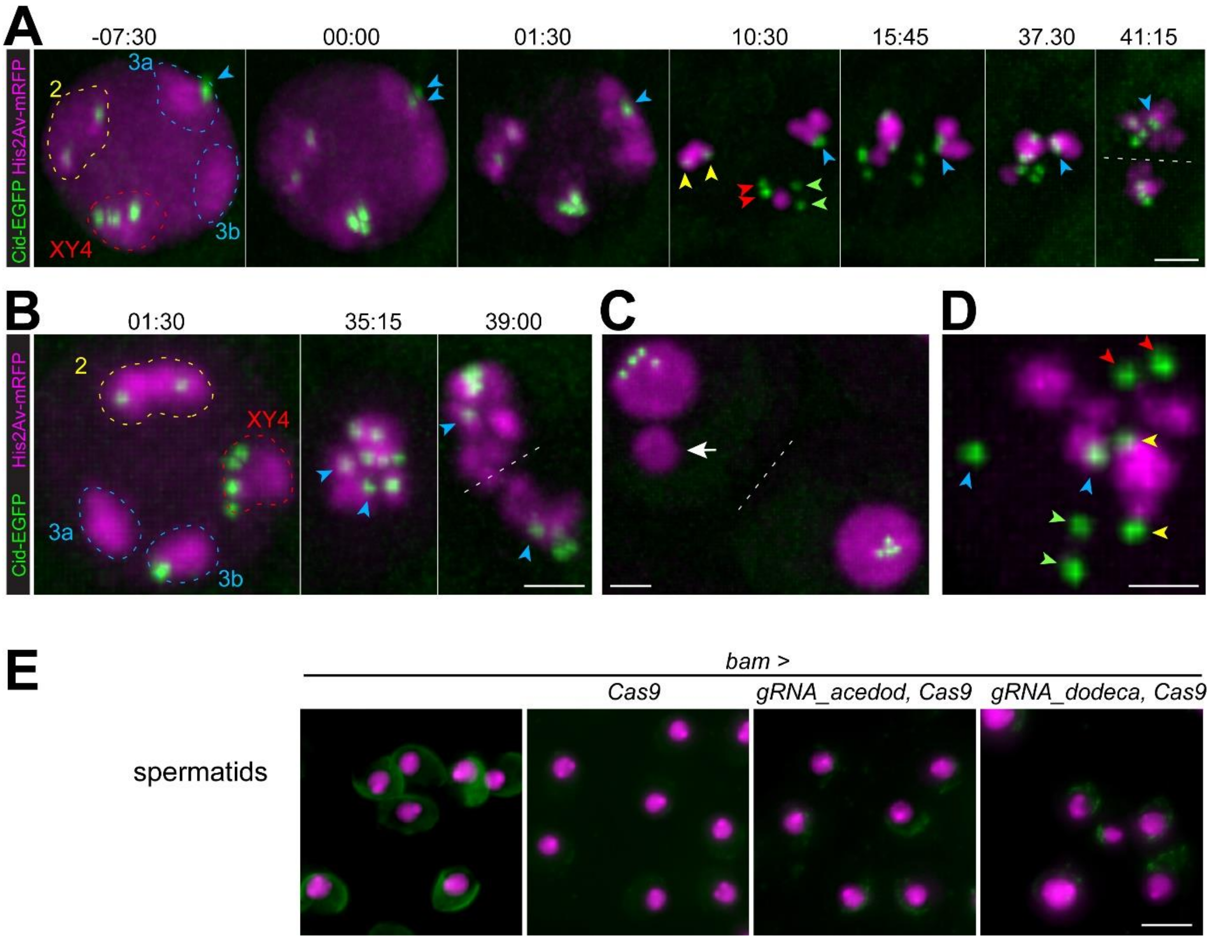

Fig. Defective chr3 segregation during meiotic divisions after dodeca satellite targeting in early spermatocytes

(**A-D**) Spermatocytes with *bam>UAS-Cas9 UAS-gRNA_dodeca* and the marker transgenes *His2Av-mRFP* and *Cenp-A/cid-EGFP* were analyzed by time-lapse imaging.

(**A**) Still frames illustrate progression through M I. Time is given relative to the onset of NEBD I. Chromosome territories before NEBD I are indicated (colored dashed lines). Colored arrowheads indicate centromeres of chr3 (blue), chr2 (yellow), chr4 (green) and chrXY (red). During anaphase (41:15), the equatorial plane (dashed line) indicates co-segregation of the two closely spaced chr3 centromeres to the upper pole.

(**B**) Still frames with labeling as in (A). The two chr3 centromeres, while unresolved initially, were eventually separated in this cell and segregated to opposite spindle poles with chromosome bridge formation during anaphase (39:00).

(**C**) Still frame from early interphase during interkinesis revealing the formation of a micronucleus lacking Cid-EGFP dots (arrow).

(**D**) Still frame from a cell in prometaphase I. Centromere labeling as in (C) reveals a chr3 centromere (blue arrowhead on the left) far away from chromatin.

(**E**) Nuclei of early spermatids in squash preparation of testes with *bam-GAL4-VP16* (*bam>*) and the indicated UAS transgenes after labeling with anti-Lamin Dm0 and a DNA stain. The high variability of the nuclear DNA content after dodeca satellite cutting indicates meiotic chromosome segregation defects.

Scale bars = 3 µm (A-D) and 5 µm (E).

References

1. Chang C-H, Chavan A, Palladino J, Wei X, Martins NMC, Santinello B, et al. Islands of retroelements are major components of Drosophila centromeres. PLoS biology. 2019; 17:e3000241. Epub 2019/05/14. doi: 10.1371/journal.pbio.3000241 PMID: 31086362.
2. Garavis M, Mendez-Lago M, Gabelica V, Whitehead SL, Gonzalez C, Villasante A. The structure of an endogenous Drosophila centromere reveals the prevalence of tandemly repeated sequences able to form i-motifs. Scientific reports. 2015; 5:13307. doi: 10.1038/srep13307 PMID: 26289671.
3. Sun MS, Weber J, Blattner AC, Chaurasia S, Lehner CF. MNM and SNM maintain but do not establish achiasmate homolog conjunction during Drosophila male meiosis. PLoS genetics. 2019; 15:e1008162. doi: 10.1371/journal.pgen.1008162 PMID: 31136586.
