# Supplementary figures and images for: Dispersive forces and resisting spot welds by alternative homolog conjunction govern chromosome shape in Drosophila spermatocytes during prophase I
Source: PLoS Genet. 2022 Jul 27;18(7):e1010327. doi: 10.1371/journal.pgen.1010327 (PMC9359577; doi:10.1371/journal.pgen.1010327)

$T(2;3)Eip74EF^1$

Cid-EGFP

His2Av-mRFP

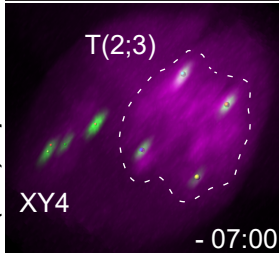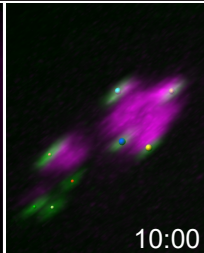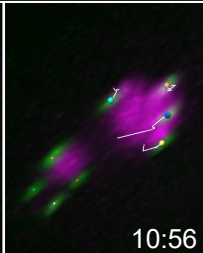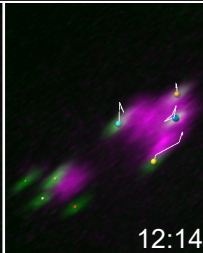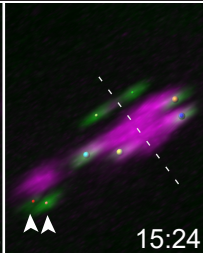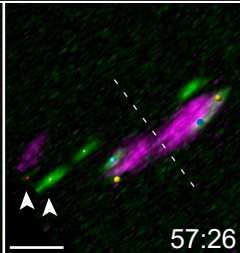

Supplement: S1 Fig — Still frames after time lapse imaging of His2Av-mRFP and Cenp-A/Cid-EGFP in spermatocytes heterozygous for T(2;3)Eip74EF1 illustrate progression through M I until anaphase onset. Time (min:sec) relative to onset NEBD I. Territories formed by the quadrivalent [T(2;3)] and by the other chromosomes (XY4) are indicated in the first frame, and equatorial plane (dashed lines) in the last two frames. Tracked centromeres are marked by spheres with colors indicating the associated chrX and chrY (red), chr4 (green), chr2 and chr3 (yellow and blue). Re-orientation of a quadrivalent centromere (dark blue sphere) occurs between 10:00 and 10:56 during prometaphase I. Centromeres of the sex chromosome bivalent (arrowheads in the last two frames) fail to attach to the distant spindle pole on the other side of the quadrivalent. Scale bar = 3 μm. (PDF) [file pgen.1010327.s002.pdf]

MNM-EGFP ◀  
Cid/CENP-A-EGFP ◀  
His2Av-mRFP

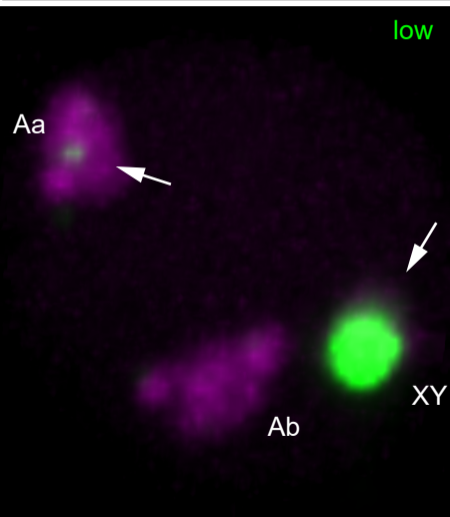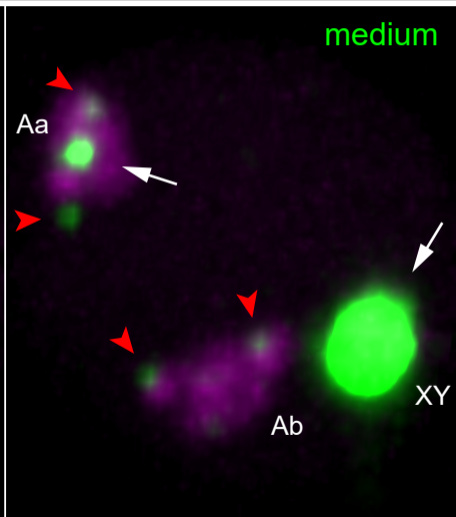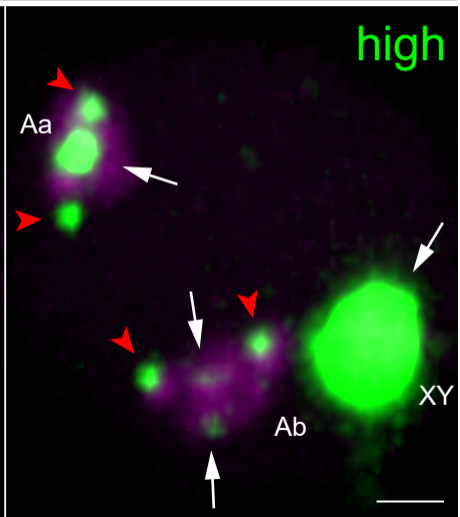

Supplement: S2 Fig — Control spermatocytes expressing His2Av-mRFP, cid-EGFP and bam>mnm-EGFP were analyzed by time-lapse imaging. The same still frame from early prometaphase I is displayed three times with increasing enhancement of green signal intensities from left to right (low, medium and high). The chrXY bivalent (XY) and the large autosomal bivalents (Aa and Ab) are indicated, as well as visible green dots representing MNM-EGFP (arrows) and centromeric Cid-EGFP (red arrowheads). MNM-EGFP dots and centromeric Cid-EGFP dots on large autosomal bivalents could be differentiated because the former but not the latter disappeared during exit from M I. Centromeric Cid-EGFP dots have comparable intensities in contrast to the highly variable MNM-EGFP dots localized at a medial position between the centromeres. The strong MNM-EGFP dot on the chrXY bivalent presumably masks Cid-EGFP signals on the bivalents with chrXY and chr4, as well as MNM-EGFP signals on the chr4 bivalent. Scale bar = 2 μM. (PDF) [file pgen.1010327.s003.pdf]
