# Supplementary material for: Dispersive forces and resisting spot welds by alternative homolog conjunction govern chromosome shape in Drosophila spermatocytes during prophase I
Source: PLoS Genet. 2022 Jul 27;18(7):e1010327. doi: 10.1371/journal.pgen.1010327 (PMC9359577; doi:10.1371/journal.pgen.1010327)
Supplement: S3 Fig — Progression through M I was analyzed by time-lapse imaging of spermatocytes with His2Av-mRFP and bamP-GAL4-VP16 driving expression of either only UASt-EGFP-Cap-H2 (top row) or both UASt-EGFP-Cap-H2 and UASt-mnm-EGFP (middle and bottom rows). bam> EGFP-Cap-H2 precludes the pairing of autosomal homologs, which normally occurs in early spermatocytes. Protein instability of EGFP-Cap-H2 after transient bamP-GAL4-VP16 driven expression in early spermatocytes results in an absence of EGFP-Cap-H2 signals during M I (top row). In contrast, MNM-EGFP dots (middle and bottom row) perdure until M I. Beyond the strong MNM-EGFP dots on the chrXY bivalent (XY), autosomal univalents displayed weaker signals (arrows) of variable intensities (arrow size) comparable to control spermatocytes (Fig 6A). Still frames from spermatocytes progressing through M I are displayed in the top and middle rows. Time (min:sec) relative to onset NEBD I. The bottom row displays additional examples of prometaphase I spermatocytes. Scale bars = 2 μM. (PDF) [file pgen.1010327.s004.pdf]

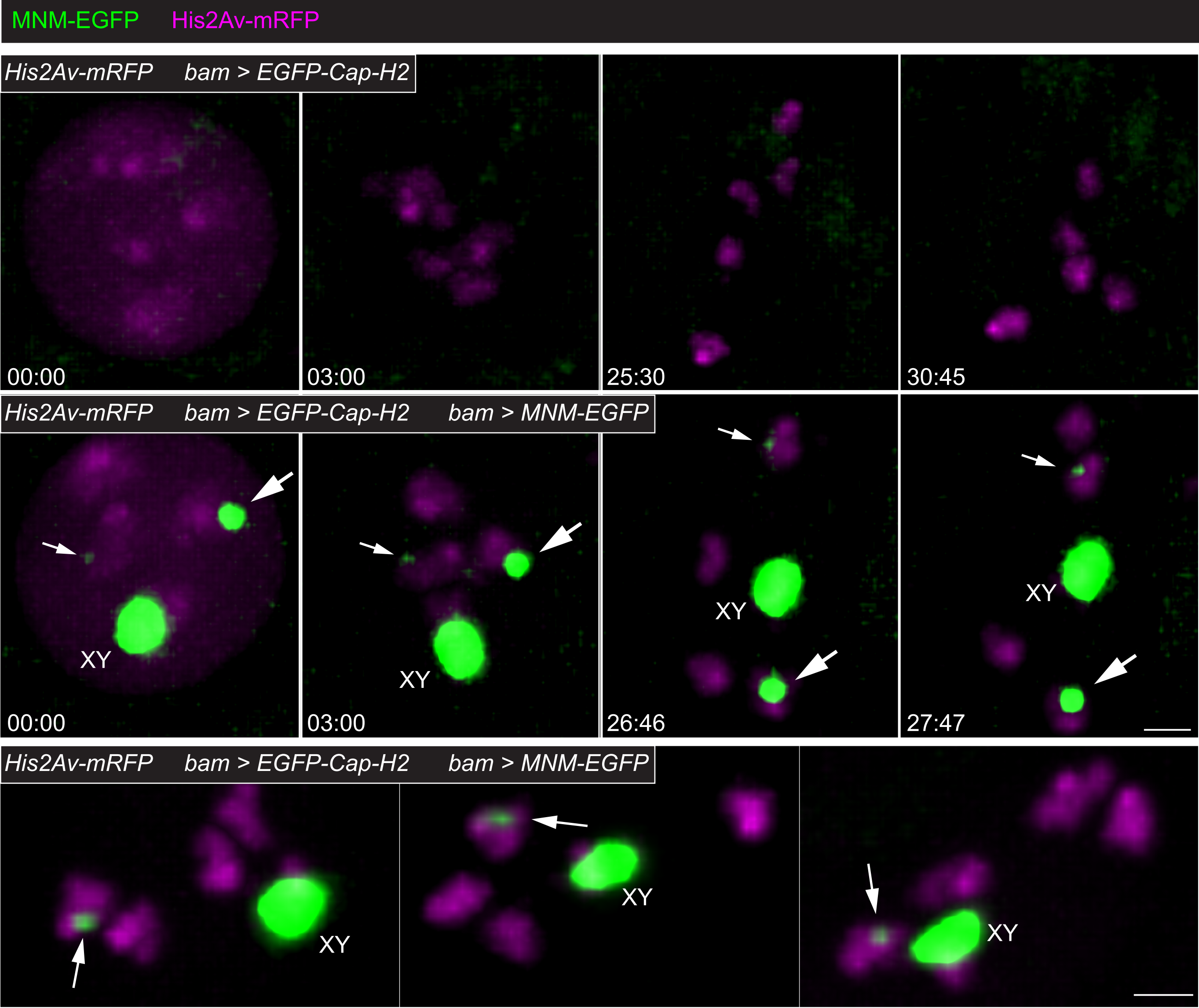
MNM-EGFP
His2Av-mRFP
His2Av-mRFP
bam > EGFP-Cap-H2
00:00
03:00
25:30
30:45
His2Av-mRFP
bam > EGFP-Cap-H2
bam > MNM-EGFP
XY
00:00
XY
03:00
XY
26:46
XY
27:47
His2Av-mRFP
bam > EGFP-Cap-H2
bam > MNM-EGFP
XY
XY
XY
